# Predictive models of eukaryotic transcriptional regulation reveals changes in transcription factor roles and promoter usage between metabolic conditions

**DOI:** 10.1093/nar/gkz253

**Published:** 2019-04-12

**Authors:** Petter Holland, David Bergenholm, Christoph S Börlin, Guodong Liu, Jens Nielsen

**Affiliations:** 1 Department of Biology and Biological Engineering, Chalmers University of Technology, Gothenburg SE-41296, Sweden; 2 Novo Nordisk Foundation Center for Biosustainability, Chalmers University of Technology, Gothenburg SE-41296, Sweden; 3 Novo Nordisk Foundation Center for Biosustainability, Technical University of Denmark, Kgs. Lyngby DK-2800, Denmark

## Abstract

Transcription factors (TF) are central to transcriptional regulation, but they are often studied in relative isolation and without close control of the metabolic state of the cell. Here, we describe genome-wide binding (by ChIP-exo) of 15 yeast TFs in four chemostat conditions that cover a range of metabolic states. We integrate this data with transcriptomics and six additional recently mapped TFs to identify predictive models describing how TFs control gene expression in different metabolic conditions. Contributions by TFs to gene regulation are predicted to be mostly activating, additive and well approximated by assuming linear effects from TF binding signal. Notably, using TF binding peaks from peak finding algorithms gave distinctly worse predictions than simply summing the low-noise and high-resolution TF ChIP-exo reads on promoters. Finally, we discover indications of a novel functional role for three TFs; Gcn4, Ert1 and Sut1 during nitrogen limited aerobic fermentation. In only this condition, the three TFs have correlated binding to a large number of genes (enriched for glycolytic and translation processes) and a negative correlation to target gene transcript levels.

## INTRODUCTION

The relationship between transcription factor (TF) binding to DNA and gene transcription in eukaryotes is complex. This is highlighted in several studies integrating chromatin immunoprecipitation (ChIP)-based TF binding data with transcriptomics from knockout or knockdown experiments of the TF with the goal of defining regulatory targets. Studies of transcriptional response to hormones found that in mice with the TF glucocorticoid receptor knocked out, only 11% of the differently expressed genes were targeted by the TF (1) and a similar study of human estrogen receptor function found only 6% of differentially expressed genes to be targeted (2). Integration of a large-scale study using microarray transcriptomics in yeast TFs deletion strains (3) with previously generated ChIP-chip data (4) for 188 TFs showed even less overlap with an average 3% of differentially expressed genes being targeted by the corresponding TF (5). Thus, combining ChIP methods with transcriptomics to understand transcriptional regulation in eukaryotic systems gives disappointing results compared to the demonstrated successes of this approach in bacteria (6).

The increased difficulty in understanding eukaryal gene regulation in comparison to bacteria may be explained by the additional levels of regulation present, such as nucleosome–TF interactions, histone modifications and long-range effects of binding. The impressive ENCODE dataset containing TF binding, transcriptomics as well as other chromatin features (7) has been used to explore the contributions of different features of human promoters to gene regulation. Using machine learning approaches, strong predictive models were created and analysis of the models suggested a highly interconnected regulatory system where TF binding has functional interactions with both nucleosome occupancy and histone modifications to regulate transcriptional outcomes (8). A different approach to create predictive models of transcriptional regulation based only on TF binding was to build a model from TF-association scores that includes both the strength of the binding event and the distance from a given gene in data collected from mouse embryonic stem cells (9). For 12 TFs, these scores were combined into principal components and using linear regressions on the principal components it was possible to predict an impressive 65% of the variation in gene expression genome wide (9). Such complex analysis methods will be important tools to fully understand eukaryal transcriptional regulation and may allow cell engineering relying on predictably changes in gene transcription.

While binding has been mapped for most central yeast TFs in one of the impressive large-scale studies (4,10–12), the majority of this data is captured only in a single state of the cell; exponential growth in nutrient excess. Here we performed a large-scale study of mapping TF binding of multiple yeast TFs known to be involved in metabolic regulation by ChIP-exo (chromatin immunoprecipitation with lambda exonuclease) in four distinct metabolic states of the yeast cell. We integrate TF binding data with transcriptomics of the same metabolic conditions with the goal of building predictive models using relatively simple statistical methods that allow full transparency for insights into contributions of different TFs to gene expression. Using ChIP-exo allowed us to study TF binding with high resolution and minimal background and using yeast as a model organism allowed us to study metabolic gene regulation utilizing a variety of nutrients with a constant growth rate in chemostats.

## MATERIALS AND METHODS

### Yeast strains and media

The host strain for all experiments contained in this study was *Saccharomyces cerevisiae* CEN.PK 113-5D (URA-). CEN.PK 113–5D with *Kluyveromyces lactis* URA3 (KiURA3) re-integrated was used as control strain for transcriptome analysis. Strains for ChIP-exo were created by amplifying either a TAP tag or a 9xMyc tag with KiURA3 and homology arms for recombination into the C-terminal end of the TF coding sequence.

The components of the chemostat media that were different between the experimental conditions are as follows: Nitrogen limited media – 1 g/l (NH_4_)_2_SO_4_, 5.3 g/l K_2_SO_4_, 150 ml/l glucose 40%, 12 drops Antifoam204. Ethanol limited media – 5 g/l (NH_4_)_2_SO_4_, 6.67 ml/l Ethanol 96%, 12 drops Antifoam204. Respiratory glucose limited media – 5 g/l (NH_4_)_2_SO_4_, 18.75 ml/l glucose 40%, 12 drops Antifoam204. Anaerobic glucose limited media – 5 g/l (NH_4_)_2_SO_4_, 25 ml/l glucose 40%, 4 ml/l ergosterol in Tween80 (2.6 g/l), 16 drops Antifoam204. In addition to the previously stated components changing between the media, all media have the following: 14.4 g/l KH_2_PO_4_, 0.5 g/l MgSO_4_, 1 ml/l of 1000× vitamin and 1000× trace metal stock solutions. The 1000× stocks contains the following: Vitamins – 0.05 g/l biotin, 0.2 g/l 4-aminobenzoic acid, 1 g/l nicotinic acid, 1 g/l calcium pantothenate, 1 g/l pyridoxine HCl, 1 g/l thiamine HCl, and 25 g/l myo-inositol. Trace metals – 15.0 g/l EDTA-Na_2_, 4.5 g/l ZnSO_4_·7H_2_O, 0.84 g/l MnCl_2_·2H_2_O, 0.3 g/l CoCl_2_·6H_2_O, 0.3 g/l CuSO_4_·5H_2_O, 0.4 g/l Na_2_MoO_4_·2H_2_O, 4.5 g/l CaCl_2_·2H_2_O, 3 g/l FeSO_4_·7H_2_O, 1g/l H_3_BO_3_ and 0.1 g/l KI. pH of the media was adjusted by adding KOH pellets to get media pH of 6.0–6.5 that result in a final pH of all chemostat cultures close to 5.5.

### Chemostat cultivation

Cells were cultivated in chemostats with a dilution rate of 0.1 h^−1^ at 30c. Stirring and aeration was performed by either N2 (fermentative glucose metabolism) or pressurized air (for the three other conditions) supplied to the cultures (13). Cultures were sampled for either ChIP-exo or transcriptomics after steady state was achieved for 48–60 h.

### ChIP-exo

When chemostat cultures were measured to be stable for 48–60 h, formaldehyde with a final concentration of 1% (wt/vol) and distilled water were added to the cultures to create a final OD_600_ of 1.0 and a total volume of 100ml. Cells were incubated in formaldehyde for 12 min at room temperature followed by quenching by addition of l-glycine to a final concentration of 125 mM. Cells were then washed twice with cold TBS and snap-frozen with liquid N_2_. ChIP-exo was then performed according to a protocol based on the originally established protocol (14) with certain modifications, as described in (15). Presentation of the ChIP-exo raw data and replicates is included in Supplementary Data 1.

### Peak finding and target gene identification

Peak detection was performed by GEM (16) with default parameters. A peak signal threshold of >2-fold peak signal over the local genomic noise was applied and peaks were annotated to a gene if it was found within –500 to +500 bp of a given genes TSS, as defined by (17). The full list of peaks detected by GEM (without peak signal threshold) for each TF is included in Supplementary Data 2.

### RNA sequencing

From chemostats at steady-state, 10 OD_600_ from three biological replicates were collected into tubes and put directly on ice. Cells were washed twice in cold TBS and snap-frozed in liquid N2. RNA extraction was performed as described in the manual for the RNeasy^®^ Mini kit (QIAGEN). RNA quality was inspected by Nanodrop, Qubit and Bioanalyzer before proceeding with sample preparation for Illumina sequencing and following sequencing on the NextSeq 500 system (2 × 75 bp, mid-output mode; Illumina). The RNA sequencing read counts per gene in each metabolic condition is included in Supplementary Data 3.

### Sequencing data processing

For both the ChIP-exo data and transcriptomics, the raw sequencing output (.fastq) was mapped to a recently published CEN.PK genome (18) using Bowtie2 (19) with the -U parameter. Samtools (20) was then used to generate sorted and indexed .bam files by first creating .bam files by the ‘view' command with parameters –bS –q 20 and further the ‘sort' and ‘index' commands.

For the ChIP-exo data, the read count covering each nucleotide position genome-wide was determined from .bam files using the genomecov function of BEDTools (21), where a read length of 10 was used for all TFs. The read counts of biological duplicate were then averaged and output as .wig files that can be found in our Mendeley Data archives as described under the Data Availability section. All further processing of ChIP-exo data is through import of .wig files into R (22) and executable scripts to reproduce all figures are included in Supplementary Data 6. We also supply in Supplementary Data 4 processed versions of the .wig data, containing only the total TF read counts for each gene promoter (TSS –500 to TSS +500 bp) for each metabolic condition. These files can be used directly as inputs to replicate our linear regression analysis.

For RNA sequencing data analysis, Subread (23) was used to map the aligned reads (.bam) to gene annotations. The resulting output of transcript read counts for the replicates from Subread (171206_CENPK_subOut_wt.txt) can be found in Supplementary Data 3. Using R (22), genes were first filtered to only include those with minimum 1 read detected in all samples and then the edgeR (24) package was used to calculate FPKM values before averaging the triplicates. All subsequent analysis using the transcriptomics data in the manuscript can be reproduced using the R scripts supplied in Supplementary Data 6.

### Linear regressions

The mathematical expression of a simple linear regression using one explanatory variable (TF binding of a single TF in this case) to model the relationship to the dependent variable (FPKM transcript level in our case) is *Y_i_* = β_0_ + β_1_*X*_1*i*_ + ϵ_*i*_. *Y*_*i*_ is the FPKM of gene *i*, β_0_ is the intercept (common to all genes in the regression), *X*_1*i*_ is the amount of TF binding to gene *i*, β_1_ is the coefficient that is selected to give the best fit together with the intercept to the transcript levels (common to all genes in the regression) and ϵ_*i*_ is the error of the prediction for gene *i*.

In multiple linear regressions, several predictors are added together, each with their own coefficient. The equation then takes the format: *Y_i_* = β_0_ + β_1_*X*_1*i*_ + … + β_*k*_ X_*ki*_ + ϵ_*i*_ where in our case k indicates the index of a TF. While our analysis using TF binding contains 21 TFs, we always use variable selection (TF selection) from the earth package (25) in R for multiple linear regressions, in which only the most predictive set of TFs will be included and added together for predictions of transcript levels of a given set of genes. In some of our analysis we also allow the earth scripts to introduce splines (earth() parameters ‘linpreds = F’ and ‘endspan = 100’), effectively allowing the algorithm to model regions where it is advantageous to have nonlinearities in the explanatory variables.

## RESULTS

### Most yeast metabolic TFs show large changes in genes targets in different metabolic states

To get information about several distinctly different states of yeast metabolism we decided to analyze gene expression regulation in chemostat cultures operated at the same specific growth rate, but still causing a range of different types of metabolism; aerobic fermentation using nitrogen limitation, respiratory glucose metabolism using glucose limitation, fermentative glucose metabolism using anaerobic conditions, and gluconeogenic respiration using ethanol limitation. These four states of metabolism should involve large changes in central carbon metabolism and hence we focused on TFs that have enriched binding (relative to all other binding targets) to central carbon metabolism enzymes. To define a list of TFs to focus on we started from the landmark dataset collected by Harbison *et al.* containing TF promoter enrichment genome-wide for a majority of yeast TFs mapped by ChIP-chip in batch cultures with rich media (4). All TFs that had >50 total targeted genes and >5% enrichment of central carbon metabolism genes were selected as candidates, as well as certain additional TFs suggested from other studies to be important for controlling central carbon metabolism. The criteria and process of selecting TFs for this study is described in more detail in Supplementary Data 5.

To map and quantify TF binding, strains were created with TFs tagged by a C-terminal TAP or 9xMyc tag. All strains were validated for presence of the tag as well as functional binding of the tagged TF to a known target gene's promoter by ChIP-qPCR. The successfully validated strains were cultivated as biological duplicates in the four different chemostat conditions and genome-wide binding events were mapped and quantified by ChIP-exo. This method is an improvement over ChIP-seq, including exonuclease treatment of the cross-linked TF-DNA complex to increase the resolution and reduce unspecific background binding (14). A demonstration of our raw data and replicates is shown for each TF in Supplementary Data 1.

**Figure 1.**
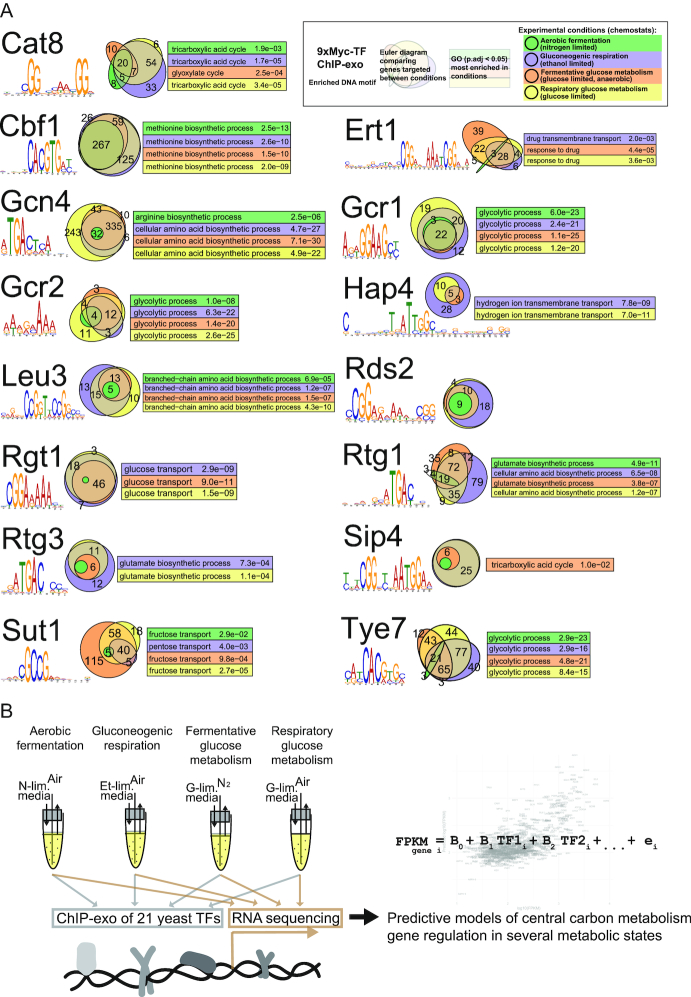
(**A**) Genome-wide TF binding characterization for multiple conditions of 15 TFs first described in this study. The most enriched DNA sequence (defined by MEME) is indicated under the TF name. The euler diagrams indicate the number of genes targeted between the experimental conditions and how the conditions overlap. Numbers are shown for all overlaps with more than two genes. For each condition, the top significant (if there is any with *P*.adj < 0.05) GO category for the genes targeted that condition is shown in the same color color of the condition in the euler diagram. (**B**) A summary of our experimental approach.

Peaks were identified by GEM (16), the duplicates averaged, and a signal threshold of >2 peak signal relative to the noise of the local genomic context was applied. Comparing to what degree the targeted genes overlap between the experimental conditions (Figure 1A), only two of the 15 TFs first reported in this study show a relatively stable set of targets between the studied nutrient limited conditions: Cbf1 and Gcr1. For the remaining TFs there are large changes in which genes are being targeted between conditions. By analyzing the targeted genes for the most relatively enriched gene ontology (GO) term, we confirm many well-known metabolic roles for these TFs, but also find indications for new functions such as drug transport for Ert1 and carbohydrate transport for Sut1 (Figure 1A).

For further analysis of the relationship between TF binding and transcriptional outcomes, we combined binding information of the 15 TFs reported here with data for an additional 6 TFs obtained with the same experimental conditions and using the same protocols (Ino2, Ino4, Hap1, Oaf1 and Pip2 from Bergenholm *et al.* (26) and Stb5 from Ouyang *et al.* (27) (Supplementary Figure S1A). We first explored the general distribution of peaks on promoters relative to the TSS and we found the expected strong enrichment upstream of the TSS for all metabolic conditions (Supplementary Figure S1b). Notably, when comparing the number of peaks detected for each condition, we discovered a significant decrease in count of peaks for most of the studied TFs in aerobic fermentation (Supplementary Figure S1C).

To look for an explanation for the broad changes in which genes are targeted between conditions we compared the most enriched DNA motif bound by the TFs for the different metabolic conditions. For most TFs, we found only minor differences in the enriched motif between conditions (Supplementary Figure S2A). There are a few notable situations where the most enriched motif may be different in one condition, for example Gcn4 and Rtg1 in aerobic fermentation, but we cannot conclude if these cases are from a true change of DNA preference for the TF or if it is due to noisy variation from the TF having fewer peaks in aerobic fermentation. For the lipid metabolism TFs Ino2, Ino4, Hap1, Oaf1 and Pip2, this analysis was performed in Bergenholm *et al.* (26), with similar conclusions.

Our data and peak detection was further compared to two previously published datasets. We focused on eight TFs that are first reported in this manuscript where binding in multiple conditions was reported by Harbison *et al.* (4) and we also compared to the refined peak definitions reported in the MacIsaac *et al.* (28). We see strong overlap for many TFs and generally stronger overlap with the Harbison *et al.* experiments using synthetic media (Supplementary Figure S2B), an expected observation because this media more closely resembles our experimental conditions. Two TFs show relatively poor overlap with existing datasets, Rtg1 and Rtg3, but we do see a strong enrichment of both TFs to genes involved in amino acid biosynthesis (Figure 1A), which is a role for these TFs that is thoroughly demonstrated (29,30) and makes us confident in the quality of our data also for these TFs.

While the selection of TFs was focused on finding TFs enriched for binding to central carbon metabolism genes, we decided to expand the gene sets for further studies of how the TFs are affecting transcriptional regulation to cover all metabolic genes. Metabolic genes were defined as being included in the latest published yeast genome-scale model, v7.6 (31); in total 849 genes from the model that have a clearly defined TSS (17) and where we also have robust gene expression data from transcriptomics were selected for further analysis. Using all metabolic genes was a compromise to have enough genes for strong statistical power and reliable observations from predictive models, but also retain the property of having relatively good TF-coverage of the genes. Our experimental approach is summarized in Figure 1B.

### Comparing predictive models of transcriptional regulation

**Figure 2.**
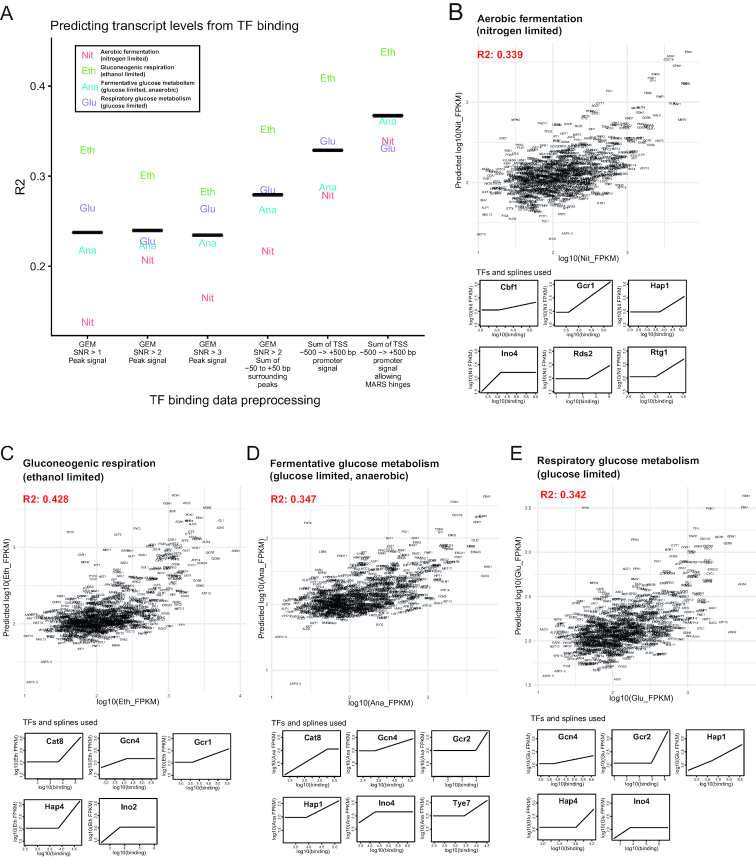
Comparing performance of TF binding data preprocessing in linear regressions to predict transcript levels and details of multivariate adaptive regression splines (MARS) models. (**A**) Correlations between predicted transcript levels and real transcript levels for the different formats of TF binding data. The black line indicates the mean of the four metabolic conditions. (**B**–**E**) MARS used to predict metabolic gene transcript levels of the different conditions from the amount of TF binding per gene promoter. The boxes shown below the predictions plots represent the different TFs that are selected by MARS to give strongest predictive performance in the conditions and how their signal is contributing to predictions in the model.

We next compared performance of different types of preprocessing of the TF binding data in predicting transcript levels (measured by RNA sequencing) using multiple linear regressions. The regressions assume a linear relationship between TF binding and effects on transcriptional regulation and we build a model where TFs binding signal is multiplied by a coefficient and added together to predict transcript levels. We first tested different signal/noise ratio (SNR) thresholds for TF peak binding signal, but found only a minimal effect on performance of the predictive models (Figure 2A). A different numeric representation of TF binding is to sum TF binding over an interval of DNA and we found that summing all binding -50 to +50bp around the identified peaks gave stronger predictive power to transcriptional outcomes (Figure 2A). We further tested an even simpler summation of the whole promoter region and found that this gave even better predictive power (Figure 2A). We think this improvement is most likely driven by contributions to transcriptional regulation from relatively weaker TF binding events that are not strong enough to be detected by a peak finding algorithm. The reduced background of ChIP-exo is here leveraged to be able to detect such weaker events over background noise. The promoter signal sum data format was also tested with multivariate adaptive regression splines (MARS) (32). In MARS, if it is advantageous for prediction performance, the algorithm can introduce splines in the linear regressions, effectively allowing a type of peak definition where the peak threshold (spline) is introduced to create a linear relationship between TF binding and transcript levels only for a certain range of TF binding strength. We found that with MARS, the performance of the predictions further increased.

We were curious to see where in the promoter region TF binding is most strongly contributing to gene regulation. We tested the predictive power of binding in segments of the promoter using linear regressions and found that binding signal upstream of the TSS (where we also detect the majority of strong TF-binding peaks, Supplementary Figure S1B) is predicted to be most consequential for transcriptional regulation (Supplementary Figure S2C), but with a notable influence also from binding directly downstream of the TSS. Comparing the conditions, it appears that there is a relative increase in influence of TF binding directly downstream of the TSS in aerobic fermentation (Supplementary Figure S2c; highest point of red line is downstream of TSS while highest point of the other conditions is upstream of TSS). To select a region of a gene's promoter which captures as much as possible of the consequential TF binding for further analysis, we started with the assumption of a symmetrical region around the TSS (assumed based on Supplementary Figure S2c) and tested extensions of this region in 50 bp increments for predicting transcript levels (Supplementary Figure S2d). The performance of predictions increase until it reaches –500 to +500 around the TSS, after which there is no further increase, indicating that this region contains a majority of the consequential TF binding.

### MARS define a set of core TFs for different conditions and reveal general quantitative features of the relationship between TF binding and transcriptional regulation

Based on the finding that MARS provided the best predictions of transcript levels from TF binding (Figure 2A), we explored what we could learn about the roles and functions of TFs from MARS regressions. For multiple linear regressions, the interesting parameters to describe TF function are the coefficients, which can tell us if the TF is an activator (positive correlation between binding and transcript levels) or a repressor (negative correlation between binding and transcript levels). The MARS algorithm can also introduce splines to improve prediction performance, which can be another parameter of TF function, describing the range of TF binding where there is a linear relationship with transcriptional outcomes. Finally, variable selection in MARS will select only the best combination of TFs to predict as much as possible while penalizing increasing the complexity of the model. To define a set of TFs that are most strongly predictive of transcriptional regulation for each condition, we used MARS with cross validation for the TF and spline selection to ensure that only the most robust TFs and splines were included in the final model (model building is illustrated in Supplementary Figure S3A). The resulting predictions of transcript levels from TF binding using MARS for the four metabolic conditions are shown in Figure 2B–E. Using these conservative MARS models, we could predict 34–43% of the variation in expression levels of metabolic genes at the four metabolic conditions we investigated. We judged this predictive power as being sufficiently good to assume that the parameters given to the TFs in these predictive models can give insights into how the TFs are contributing to gene regulation in metabolism. Typical quality control checks of the MARS models presented in Figure 2B–E, such as the distribution of residuals and QQ plots are shown in Supplementary Figure S3B.

The details of how TFs are contributing at different TF binding strengths in these predictive models are shown in the lower panels of Figure 2B–E. The coefficient of the TFs from the regressions are illustrated here by either an upward slope (activation) or a downward slope (repression). A striking observation from these models is that of the 22 TF-transcript level correlations selected by MARS over the four metabolic conditions, the linear correlations were all positive, suggesting a predominance of activation in yeast metabolic gene regulation by TFs. MARS can also create segments where there is no correlation between TF binding and transcript levels if that is advantageous for the predictions. The most common way that the MARS algorithms created splines from TF binding (15/22 cases) is to introduce a threshold after which there is a linear relationship between TF binding and transcript level. MARS also found relationships between TF binding and transcription that show a saturation effect, where more binding does not lead to higher expression (6/22 cases). Of these six cases, four of them are from the well-known interaction partners Ino2 and Ino4, suggesting that nonlinearity between binding and functional outcomes may be a general feature of this TF complex.

### Contributions from several TFs on gene regulation are generally additive, but exploring collinear TFs indicates cases of more complex functional interactions

**Figure 3.**
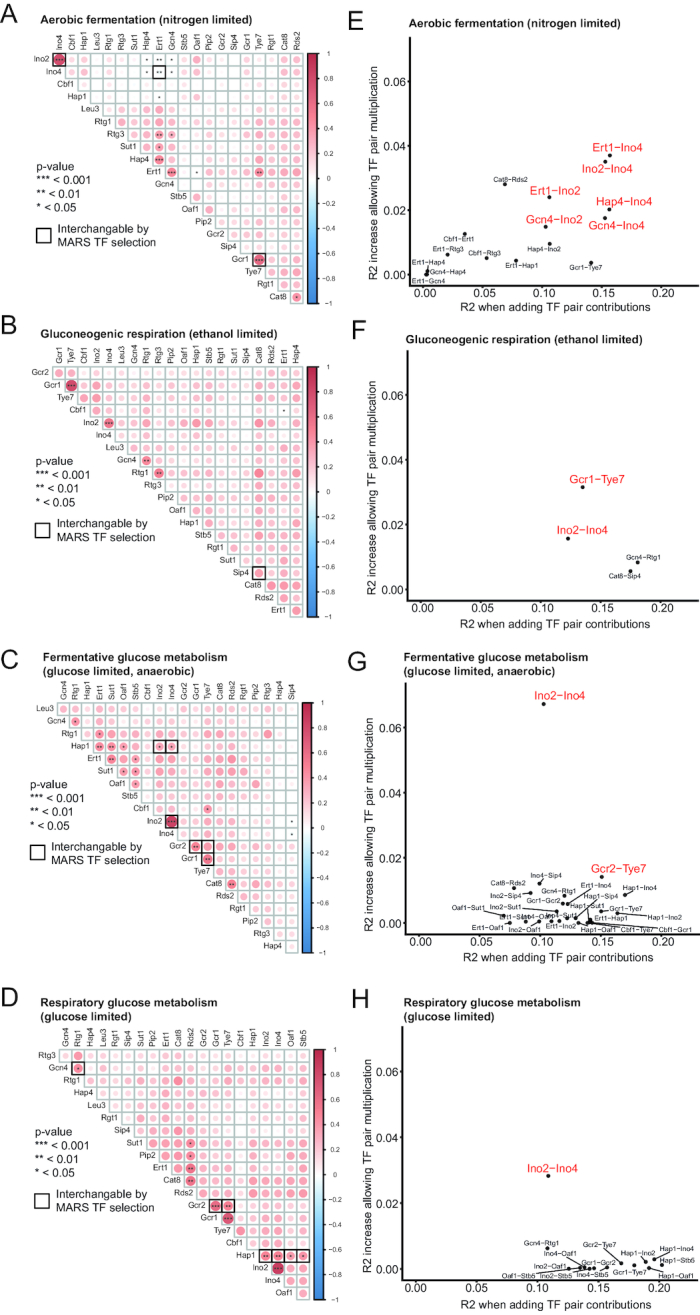
Exploring contributions of collinear TF pairs to transcriptional regulation. (**A**–**D**) Correlation plots illustrating Pearsons correlations (in color) between TF binding in promoters of metabolic genes. Significance (Pearson's product moment correlation coefficient) is illustrated for TF pairs with *P* < 0.05, by one or several asterisks, as indicated. Pairs of significantly collinear TFs that are interchangeable in the MARS TF selection in Figure 2B–E are indicated by a stronger border in (A–D). (E–**H**) Linear regressions of collinear TF pairs were tested with and without allowing a multiplication of TF signals of the two TFs. TF pairs indicated in red and with larger fonts have an *R*^2^ of the additive regression >0.1 and increased performance with including a multiplication of the TF pairs of at least 10%.

In multiple linear regressions, correlation between explanatory variables (here TFs) leads to multicollinearity, a redundancy in the information contained in explanatory variables which may complicate the interpretation of the model, In our data, collinearity between TFs could be due to TFs interacting in a common protein complex, or responding independently to the same cellular cues that regulate TF-DNA binding. A feature of the TF selection in MARS is that if there is collinearity between two TFs that are both strongly predictive of transcript levels, only the TF that gives slightly better predictions will be included while the other TF will not be visible. To look for such collinear TFs we first mapped the correlations in binding signal over metabolic genes and calculated significance of the correlations (Figure 3A–D). To find cases in the MARS models of collinearity where a TF is not included because there is a slightly better predictor selected, we tested if all included TFs could be substituted by other TFs with significantly correlated binding. Such cases are shown with black borders in Figure 3A–D and they are TF pairs predicted to regulate similar sets of genes in similar ways, indicating overlapping functions and/or possibly more complex TF–TF interactions. This analysis revealed several known cases of TF interactions such as Ino2–Ino4, Gcr1-Gcr2-Tye7 and Cat8-Sip4, but also novel potential interactions such as Gcn4-Rtg1 and Ert1-Ino4.

In the MARS models shown in Figure 2B–E, the contribution of TFs binding to each gene is multiplied by a coefficient and then added to get the final predicted transcript level for that gene. We further looked for TF-TF interactions that contribute to transcriptional regulation in ways that are numerically more complex than simple addition. All the significantly correlated TFs were tested if the multiplication of the signal of two collinear TFs give additional predictive power compared to addition of the two TFs (Figure 3E–H). Most collinear TF pairs do not show a strong improvement in predictive power by including a multiplicative interaction term, for example the mentioned potential TF interactions of Cat8-Sip4 and Gcn4-Rtg1 during gluconeogenic respiration which only gave a 3% and 4% increase in predictive power, respectively (Figure 3F, percentage improvement calculated by (multiplicative R2 increase (y-axis) + additive R2 (x-axis))/additive R2 (x-axis)). The TF pair that shows the clearest indications of having a more complex functional interaction is Ino2–Ino4, having 19%, 11%, 39% and 20% improvement (Figure 3E–H) in predictive power in the tested metabolic conditions by including a multiplication of the binding signals. TF pairs that together explain >10% of the metabolic gene variation using an only additive regression and also show minimum 10% improved predictive power when allowing multiplication are indicated in red in Figure 3E–H. For Ino2–Ino4, the strongest effect of the multiplication term is seen during fermentative glucose metabolism with 39% improved predictive power (Figure 3G). The plot for how the multiplied Ino2–Ino4 signal is contributing to the regression in this condition reveal that in the genes where both TFs bind strongest together, there is a predicted reduced activation as compared to intermediate binding strengths of both TFs, and a similar trend is seen for the Ino2–Ino4 pair for other metabolic conditions (Supplementary Figure S3c).

### Clustering metabolic genes based on their relative change in expression gives a strong enrichment of metabolic processes and improved predictive power of TF binding in linear regressions

**Figure 4.**
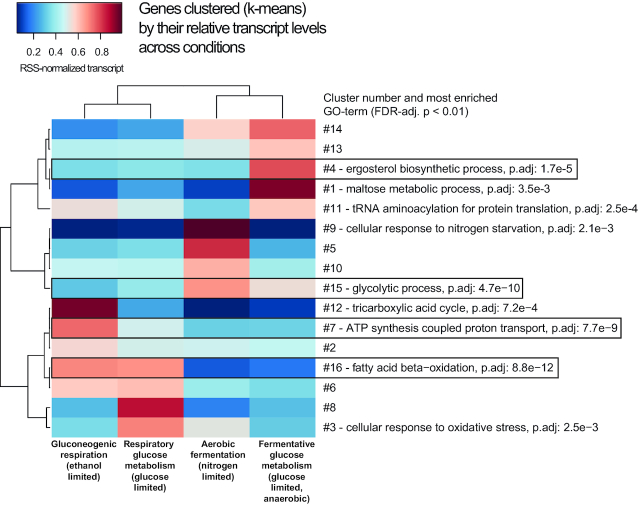
Clustering genes by their relative change in expression (sum of squares normalization) over the four experimental conditions gives enrichment of functional groups of genes. For clusters which have one or several significantly (FDR-adj *P* < 0.01) enriched GO terms, the top GO term is indicated with p.adj-value. Clusters containing central metabolic processes selected for further analysis with linear regressions in Figure 5 are indicated by a black frame.

Linear regressions of metabolic genes with TF selection through MARS defined a small set of TFs that were robustly associated with transcriptional changes over all metabolic genes (Figure 2B–E), but TFs that only regulate a smaller group of genes would be unlikely to get selected by this method. We clustered genes by their sum-of-squares normalized expression between conditions to get smaller clusters of genes with a range of gene expression levels that are appropriate for predictive modeling by multiple linear regressions. The motivation for clustering genes into smaller groups is to be able to link TFs to specific patterns of gene expression changes between the tested metabolic conditions and to functionally connected groups of genes– thus allowing more detailed predictions about the TFs’ biological roles. The optimal number of clusters to maximize the separation of the normalized expression values of metabolic genes was 16, as determined by Bayesian information criterion (Supplementary Figure S4A). Genes were sorted into 16 clusters by k-means clustering and we found that most clusters then show significant enrichment of metabolic processes, represented by GO categories (Figure 4). We further selected four clusters (indicated by black frames in Figure 4) that are both enriched for genes of central metabolic processes and have large transcriptional changes across the different metabolic conditions for further studies of how TFs are affecting gene regulation in these clusters through multiple linear regressions. While the introduction of splines was highly stable for linear regressions over all metabolic genes, we found the process of model building with MARS using splines to be less stable in smaller groups of genes (mean cluster size with 16 clusters is 55 genes). For the multiple linear regressions in the clusters, we retained TF selection (by variable selection in the MARS algorithm) to define the most important TFs, but without introduction of splines.

**Figure 5.**
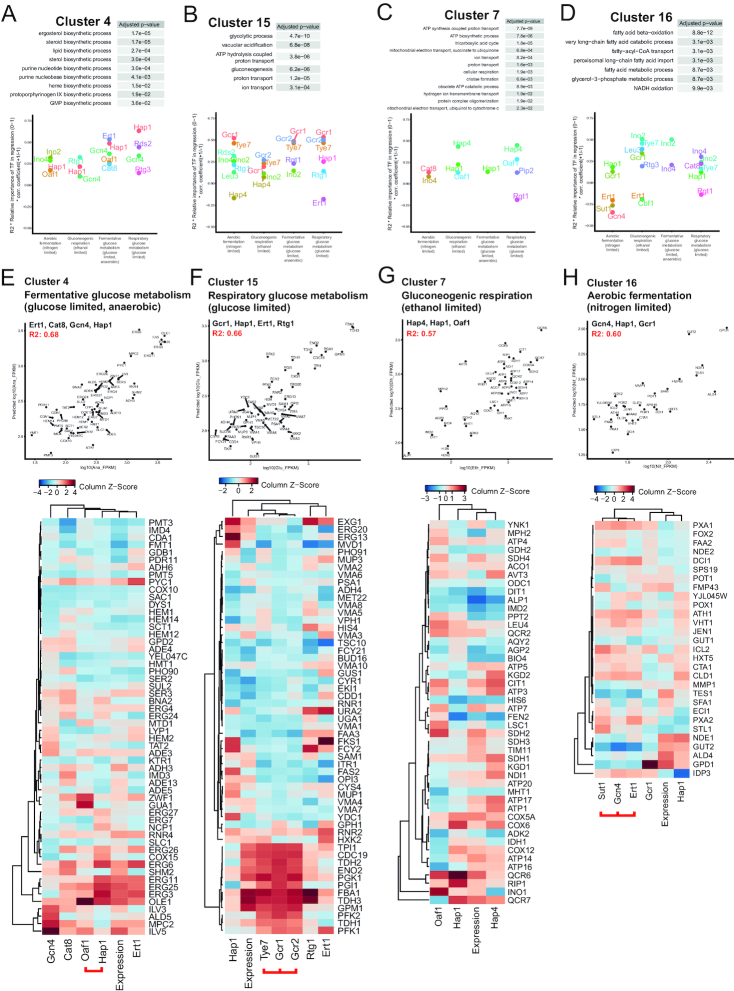
Clustering genes by relative expression gives strong predictive models of the clustered genes. (**A**–**D**) All significant (*P*.adj < 0.05) GO terms for the clustered genes and the relative importance of the TFs selected to give the strongest predictions of transcript levels for the genes in the clusters in different conditions. Linear regressions (without splines) are used and importance is calculated by R2 (of regression with selected TFs) *relative importance of each TF (0 to 1) *sign of coefficient (+1 is activation, –1 is repression). (**E**–**H**) Prediction plots showing the predicted transcript levels compared to the real transcript levels from using the selected TFs (written in subtitle of plots). R2 of predicted transcript levels compared to real transcript level is shown in red text. Heatmaps demonstrate the real transcript levels as well as binding signal of each TF normalized column-wise (*Z*-score). TFs linked by a red line under the heatmap have significant collinearity over the cluster genes and were demonstrated to be able replace the other(s) in the variable selection, thus having overlapping functions in regulation of genes in a given cluster.

Using this framework of multiple linear regression, predictions of transcriptional regulation on the clustered genes gives an improvement in predictive power compared to predictions of all metabolic genes (Figure 5E–H, R2: 0.57–0.68). To compare the importance of different TFs for the predictions of transcript levels in the groups over different conditions, we calculate the ‘TF importance’ by multiplying R2 of the multiple linear regression predictions with the relative contribution of the TF in the linear regression (0–1, calculated by model construction algorithm) and also a coefficient for activation or repression (+1 or –1, respectively). Some TFs were found to regulate a certain process over several conditions, such as Hap1 for Cluster 4, enriched for ergosterol biosynthesis genes (Figure 5A), but Cluster 4 is generally an example of a cluster with relatively large changes in importance of different TFs for gene regulation in different conditions. To get information about the complete set of TFs regulating these clusters of genes, we also included collinear TFs that were not initially included in the variable selection, but could replace a significantly correlated TF (illustrated by a red link under the TF’s names in the heatmaps of Figure 5). For Cluster 4, Oaf1 was not selected during TF selection for this cluster and was thus not used in the predictions illustrated in the prediction plot of Figure 5E, but was included in the heatmap because it was correlated to the Hap1 binding and when excluding Hap1 from the TF selection, Oaf1 was included. Because the contribution of each TF is linear in these regressions, the heatmaps give a complete view of how each gene is predicted to be regulated by different TFs. For Cluster 4 in fermentative glucose metabolism, the main contributors to ergosterol genes (ERG27, ERG26, ERG11, ERG25, ERG3) are predicted to be Ert1, Hap1 and Oaf1 (Figure 5E).

Cluster 15 is highly enriched for glycolytic processes and across conditions we see that the TFs predicted to be most important in several conditions are the well-known glycolytic regulators Gcr1, Gcr2 and Tye7 (Figure 5B). In respiratory glucose metabolism, Gcr1, Hap1, Ert1 and Rtg1 are included by variable selection and together these TFs are able to explain 66% of the variation in the cluster (Figure 5F). Both Gcr2 and Tye7 were found to be collinear and able to replace Gcr1 if it was excluded and these three TFs together are predicted to be regulating the glycolytic genes TPI1, CDC19, TDH2, ENO2, PGK1, PGI1, FBA1, TDH3, GPM1, PFK2, TDH1 and PFK1 (Figure 5F). We next focused on Cluster 7, containing genes with relatively higher expression in the two respiratory conditions (seen in Figure 4) and most strongly enriched for genes of mitochondrial ATP biosynthesis (Figure 5C). The most important TF for predictions in this cluster for both the conditions with mostly respiratory metabolism is the well-known mitochondrial regulator Hap4 (Figure 5C). During gluconeogenic respiration, Hap4 and Hap1 are predicted to both contribute to the regulation of most electron transport chain genes such as SHD1, NDI1, COX5A, COX6, QCR6, RIP1, QCR7 and ATP14 (Figure 5G). Several subunits of the ATP synthase (ATP17, ATP1 and ATP16) as well as certain TCA cycle enzymes (KGD1, KGD2) are predicted to be regulated by Hap4 without Hap1. Oaf1 also contributes to regulation of various genes of the cluster to a lesser extent, in some cases alone, or together with Hap1 and/or Hap4. We also demonstrate the same analysis for Cluster 16, most enriched for containing genes of fatty acid beta-oxidation (Figure 5D). While most of the predicted effects of TF binding on transcriptional regulation explored thus far has been activation, from the importance of TFs in the different conditions of Cluster 16, three TFs showed negative correlation to transcriptional changes during aerobic fermentation (Figure 5D). The TF selection used three TFs – Gcn4, Hap1 and Gcr1 to predict 60% of the variation in the cluster during aerobic fermentation (Figure 5H). Ert1 and Sut1 were further included in the analysis because they were found to be collinear and able to replace Gcn4 in TF selection. Exploring the contributions of the TFs to expression levels of beta-oxidation genes in the heatmap supports an inverse relationship between Sut1-Gcn4-Ert1 binding an expression levels of several beta-oxidation genes, most notably for PXA1, DCI1, CTA1, CLD1, PXA2 and IDP3 (Figure 5H).

### The influence of TFs on gene regulation from different regions of the promoter changes between metabolic conditions

**Figure 6.**
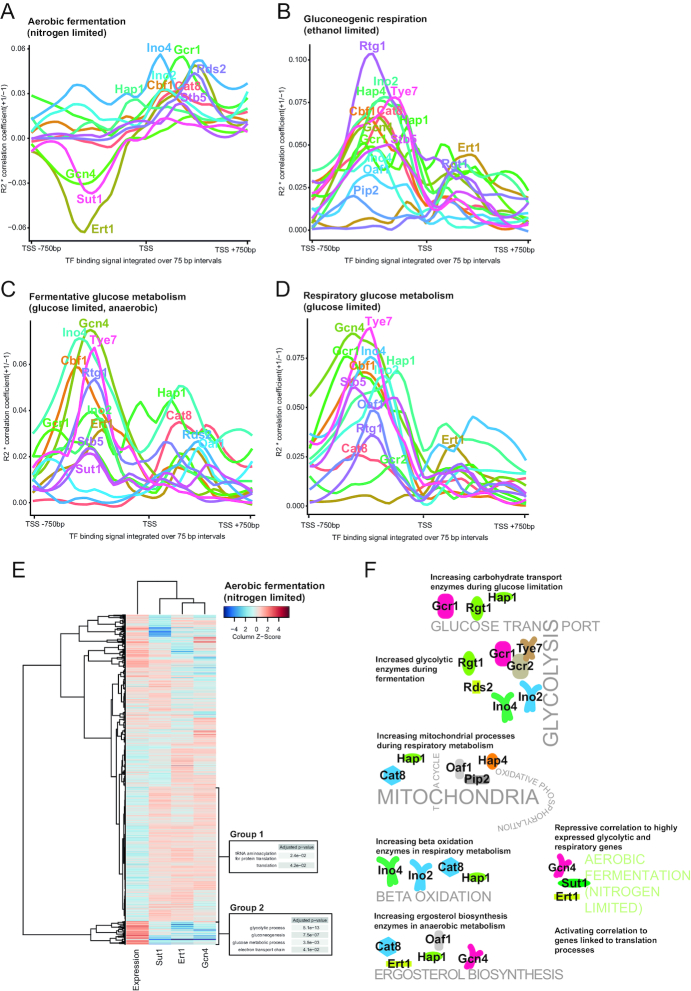
Comparing TF importance in different segments of the promoter shows a distinct pattern of TF importance during aerobic fermentation as compared to the other conditions. (**A**–**D**) Linear regressions, regressing the TF binding signal of each TF individually, in the indicated segments of the promoters, against the expression levels. Lines are labeled by the TF name near the highest absolute y-axis value. (**E**) Two groups of genes where binding of Sut1, Ert1 and Gcn4 in −450 to −250 relative to the TSS is correlated to each other and oppositely correlated to transcript levels. (**F**) Summary of the strongest predicted links between TF function and selected metabolic processes. Based on data presented in Figures 4 and 5, Supplementary Figure S4E–F and Figure 6A–E.

In the analysis shown in Supplementary Figure S2C we noticed that there were apparent differences in importance of different regions of the promoter between the conditions, most notably a predicted shift towards more consequential TF binding downstream of the TSS during aerobic fermentation. We reasoned that this could be because TFs with more consequential binding downstream of the TSS could be more important at this condition, or it could be because TFs shift their importance from one region of the promoter to another between conditions. To distinguish these two possibilities we performed simple linear regressions using the binding signal for each TF individually in 75 bp regions of the promoter to look for potential changes in TF importance in different regions of the promoter between the metabolic conditions. Comparing the resulting profiles of the explanatory power of TF binding in the experimental conditions revealed a distinctly different profile during aerobic fermentation compared to the other three metabolic conditions (Figure 6A compared to Figure 6B–D). Importantly, the differences during aerobic fermentation do not appear to be driven by one or a few TFs that regulate from downstream of the TSS with stronger importance during this condition, but rather a shift of where in the promoter several TFs are most consequential to regulation (Compare Gcr1, Ino2, Ino4, Stb5, Cbf1 in Figure 6A to B–D).

Another striking observation from the importance of TFs during aerobic fermentation was a negative correlation between transcriptional changes and binding of three TFs from an overlapping region upstream of the TSS; Sut1, Gcn4 and Ert1 (Figure 6A). These regressions were of all metabolic genes, suggesting that the negative correlation seen with these TFs on beta-oxidation genes, as seen in Figure 5H, may be a more general phenomena of aerobic fermentation. To see if these three TFs are acting on the same genes or separately we compared the binding of the three TFs in the region 250–450 upstream of the TSS together with expression levels over all metabolic genes to look for groups of genes where binding of Sut1, Gcn4 and Ert1 are correlated to each other and anti-correlated to expression levels (Figure 6E). In aerobic fermentation, we found two distinct groups of genes where all three TFs are generally correlated to each other, one with low binding of the three TFs and high expression levels and another group with relatively higher binding of all three TFs and relatively lower expression levels. Interestingly, the group of genes where the three TFs have the strongest negative correlation to transcript levels is slightly enriched for genes of translation processes (Figure 6E, Group 1), genes that are likely closely controlled due to the nitrogen limitation of these cultures. We also looked for similar relationships between binding of Sut1-Gcn4-Ert1 and transcript levels for the other experimental conditions (Supplementary Figure S4B–D), but we found no coordinated changes in the other conditions, suggesting this phenomena is specific to aerobic fermentation. We summarize some of our main findings from Figures 5 and 6A–E regarding how TFs regulate difference metabolic processes in different conditions in Figure 6F.

## DISCUSSION

The latest count of known and putative yeast TFs is 264 (33). With coverage of 21 TFs we only see part of the system of gene regulation by TFs, but by selecting the TFs most enriched to binding metabolic genes and building predictive models of metabolic gene regulation we achieved good TF coverage per gene. Through various types of analysis we discovered surprisingly large changes between the studied metabolic conditions, first encountered when comparing sets of target genes for the TFs between conditions shown in Figure 1A. To explain the changing sets of gene targets between conditions, we hypothesized that the TFs could have changes in preference of DNA motifs between different conditions. We cannot exclude changes in motif preference in certain cases such as Rtg1 and Gcn4 in aerobic fermentation (Supplementary Figure S2a), but in general the changes in motif preference between conditions are small and we think this is not a major determinant of changes in which genes are being targeted between conditions. We think the most likely alternative explanation is that there are changes in nucleosome occupancy or histone modifications that allow more or less binding to different sets of genes over the different conditions studied and that binding site preference is only partly driven by the recognized DNA motif. This idea is generally supported by the complex two-way interactions that have been suggested between TF binding and histones in eukaryotic gene regulation (8).

Based on the success of multiple linear regressions as predictive models of gene regulation from TF binding data, we propose that a large portion of transcriptional regulation by TFs in yeast is achieved from a linear effect of TF binding on transcriptional outcome. Our predictions also suggest that a large amount of the contributions to gene regulation from several metabolic TFs are additive. However, by allowing multiplication of TF binding signal, we do detect cases that indicate more complex contributions from pairs of TFs to gene regulation than a simple addition of the two TFs binding signal can capture. Most noteworthy is the relationship between Ino2 and Ino4, where the additive contribution from both TFs seem to saturate at a certain strength of binding (Supplementary Figure S3c). The Ino2–Ino4 relationship is best known for being required for phospholipid biosynthesis and Ino2 is described as most important for transcriptional activation of the targeted genes, but Ino2 also depends on Ino4 for translocation into the nucleus (34). There is additional complexity in the regulation of this complex by involvement of the Opi1 repressor, which can bind Ino2 to inhibit activation by the complex (35). We cannot conclude on why we see a saturation effect in the transcriptional activation due to increased binding of both Ino2 and Ino4, but it could be due to TF–TF competition of the complex with other components of the transcriptional machinery, or other nonlinear relationships between binding of the TFs and transcriptional outcomes. In aerobic fermentation we detect several additional TF pairs for which there may be more complex relationships than simple addition of the contributing signal. Of these, both Ert1 and Gcn4 have relationships to Ino2 and Ino4 binding where a multiplication of the binding signal clearly improves predictive power (Figure 3E). It is striking that Ert1 and Gcn4 are two of only a few TFs that show this kind of complexity seen together with the negative correlation seen between transcript levels and binding of Sut1, Gcn4 and Ert1 in aerobic fermentation, but we do not know if these observations are related.

The main advantage of using linear regressions, as compared to more complex machine learning analysis, is that each TF’s contribution in the predictions can be fully described. We highlight this feature of our analysis in Figure 5, where the groups of genes are biologically linked and small enough to illustrate the amount of binding from each selected TF in the individual genes of the clusters. In this analysis we confirm several previously demonstrated regulators of important cellular processes such as Hap1 activating ergosterol biosynthesis (36), Gcr1-Gcr2-Tye7 activating glycolytic genes (37) and Hap4 activating respiratory processes (38). We also propose previously unknown contributions from other TFs to these processes such as Ert1 activating ergosterol biosynthesis at anaerobic conditions (Figure 5E).

The minimal media used in all chemostats of this study are without added amino acids, meaning that the feedback controls activating amino acid biogenesis should generally be activated. Gcn4 is best known as an activator of amino acid biosynthesis, but also demonstrated in several studies to be a repressor of ribosomal protein expression by an unknown mechanism (39,40). The mechanistic details on how Gcn4 can activate some genes and repress others remain mostly unknown, but the repression has been linked to functional interactions with the repressive TF Rap1 and histone acetyltransferase Esa1 (41). A recent study of the yeast strain *Y. lipolytica*, describing increased lipid accumulation of this strain during nitrogen limitation, found that beta-oxidation genes were down-regulated in this condition (42). In that study, they also noted that genes with the Gcn4 motif in their promoter tended to be down-regulated during nitrogen limitation, an observation that they could not explain, but is in line with the negative correlation we observe between Gcn4 binding and transcript levels for beta oxidation genes during nitrogen limitation (Figure 5H). We propose that a similar mechanism as was observed in *Y. lipolytica* exists in *S. cerevisiae* and further extend it to affect a larger set of genes (Figure 6E) and to also involve Sut1 and Ert1. Sut1 is previously described to interact with the co-repressor complex Cyc8-Tup1 (43) while Ert1 is previously described to function as both an activator and repressor with the major described functional role being regulating genes driving the diauxic shift (44). Our data does not give any further insight into the mechanism of the Sut1-Ert1-Gcn4 repression, but based on the generally reduced number of peaks (Supplementary Figure S1C) and changes in importance of different regions of the promoter (Supplementary Figure S2C, Figure 6A–D) during aerobic fermentation, we speculate that Sut1-Ert1-Gcn4 binding is correlated to changes to the nucleosomes on each side of the TSS in certain genes, for example histone modifications or changes in nucleosome occupancy. If the changes in binding of these three TFs is not driven by changes in their preference for motifs, but instead by nucleosomes, it is also not necessarily the case that the three TFs are directly repressing – the correlation between TF binding and transcriptional regulation could potentially be a secondary effect of nucleosome changes that are also causing transcriptional changes.

In conclusion, our experimental design using chemostats to capture stable states of metabolism reveals large changes in functional roles of different TFs between metabolic states. The previously demonstrated difficulties in defining the regulatory targets of eukaryal TFs through transcriptomics after TF deletion could be partly explained by this highly dynamic nature of eukaryal TF function. If the deletion of the TF changes cellular conditions enough to shift the regulatory roles of a range of one or several other TFs, the following secondary transcriptional changes could be a source of significant changes in genes not targeted directly by the deleted TF. Our framework of using multiple linear regressions for full transparency of TF contributions to transcriptional regulation without relying on TF deletion will be equally applicable for future larger-scale studies as binding data for more TFs with condition-matched transcriptomics accumulate to gradually build a system-level understanding of eukaryotic transcriptional regulation.

## DATA AVAILABILITY

ChIP-exo raw sequencing data (.fastq) can be retrieved form Arrayexpress accession E-MTAB-6673: https://www.ebi.ac.uk/arrayexpress/experiments/E-MTAB-6673/. A processed format of the ChIP-exo data, summarized as TF reads per gene promoter is included in Supplementary Data 4. RNA sequencing raw sequencing data (.fastq) can be retrieved from Arrayexpress accession E-MTAB-6657: https://www.ebi.ac.uk/arrayexpress/experiments/E-MTAB-6657/. The number of reads annotated to each gene are found in Supplementary Data 3 and our processing of read counts to get to FPKM values that are used for all relevant analysis of this study can be reproduced through the R scripts included in Supplementary Data 6.

## Supplementary Material

gkz253_Supplemental_Files
