# Supplementary material for: Predictive models of eukaryotic transcriptional regulation reveals changes in transcription factor roles and promoter usage between metabolic conditions
Source: Nucleic Acids Res. 2019 Apr 12;47(10):4986–5000. doi: 10.1093/nar/gkz253 (PMC6547448; doi:10.1093/nar/gkz253)
Supplement: gkz253_Supplemental_Files [file gkz253_supplemental_files.zip › 190301 NAR revision SuppFig LegendsDatLeg.pdf]

Supplementary Figure 1

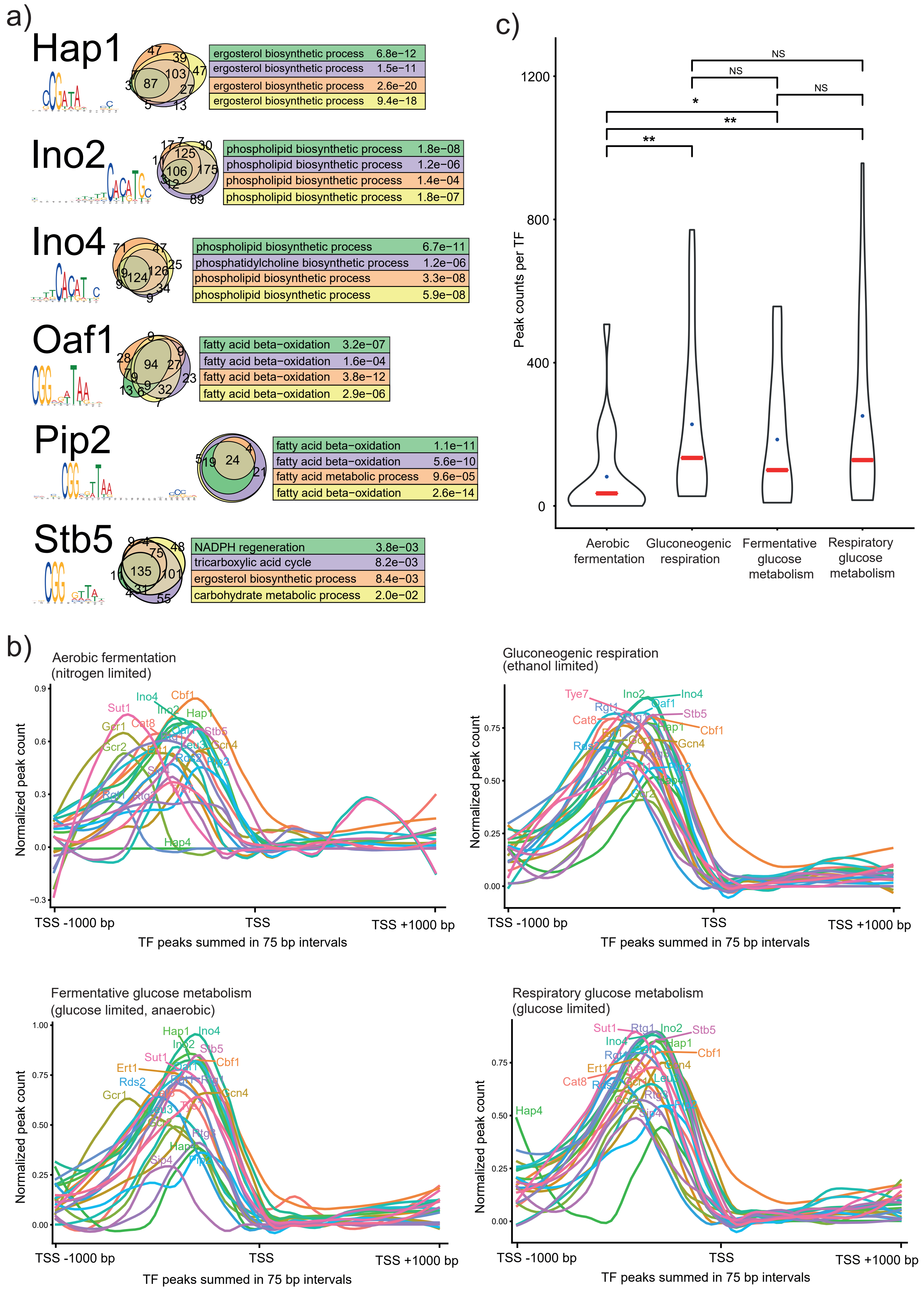

Supplementary Figure 2

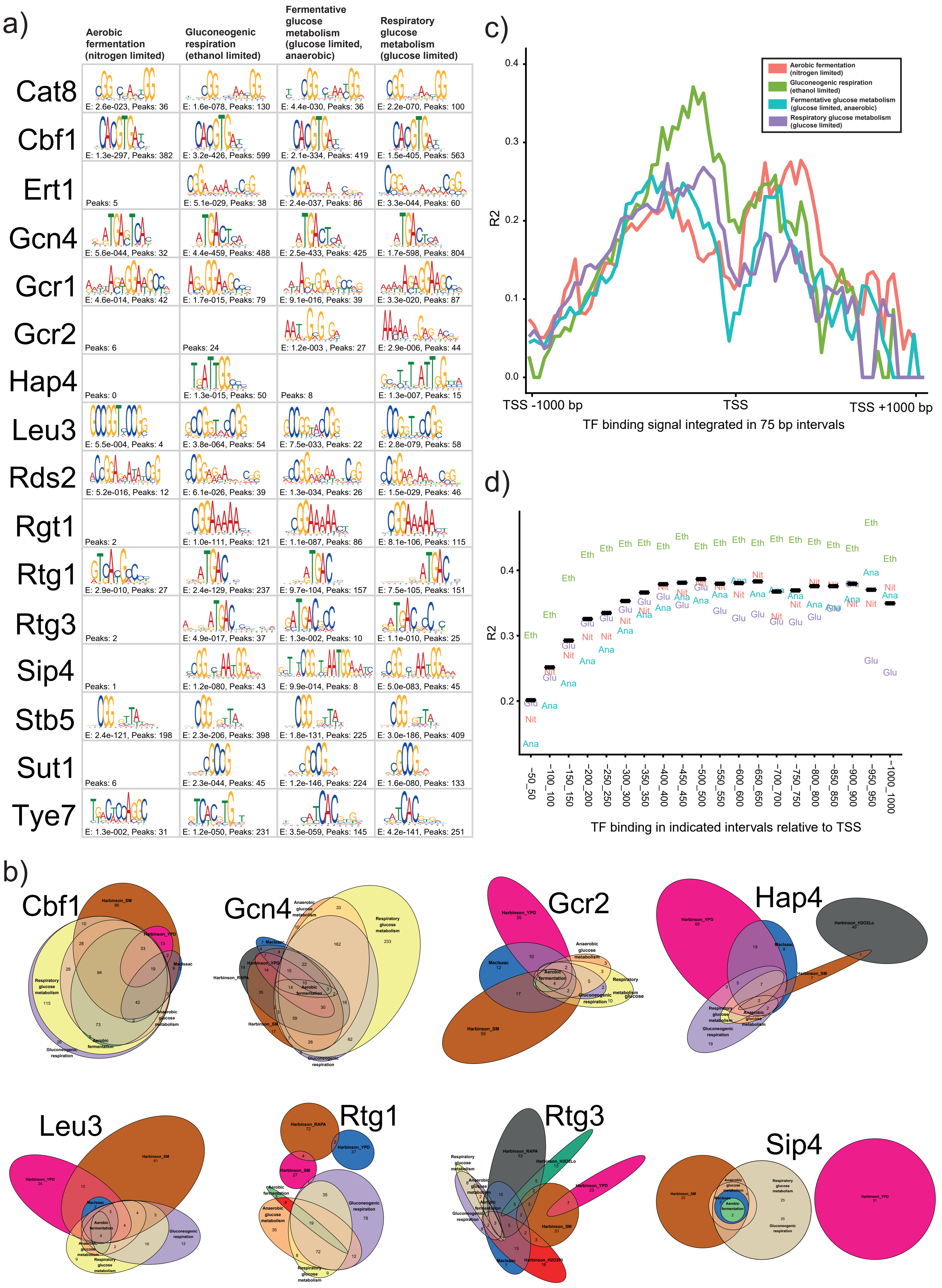

Supplementary Figure 3

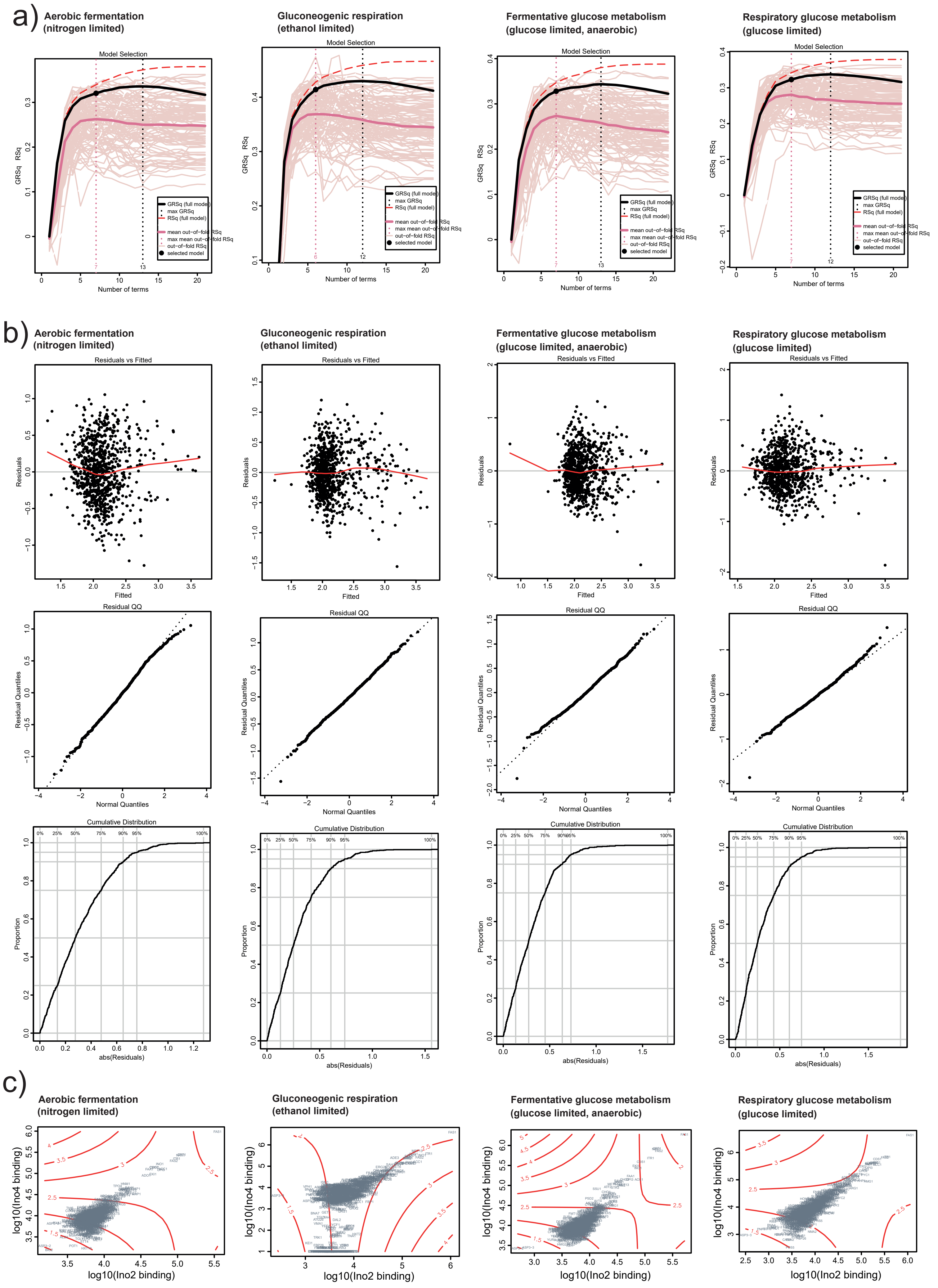

Supplementary Figure 4

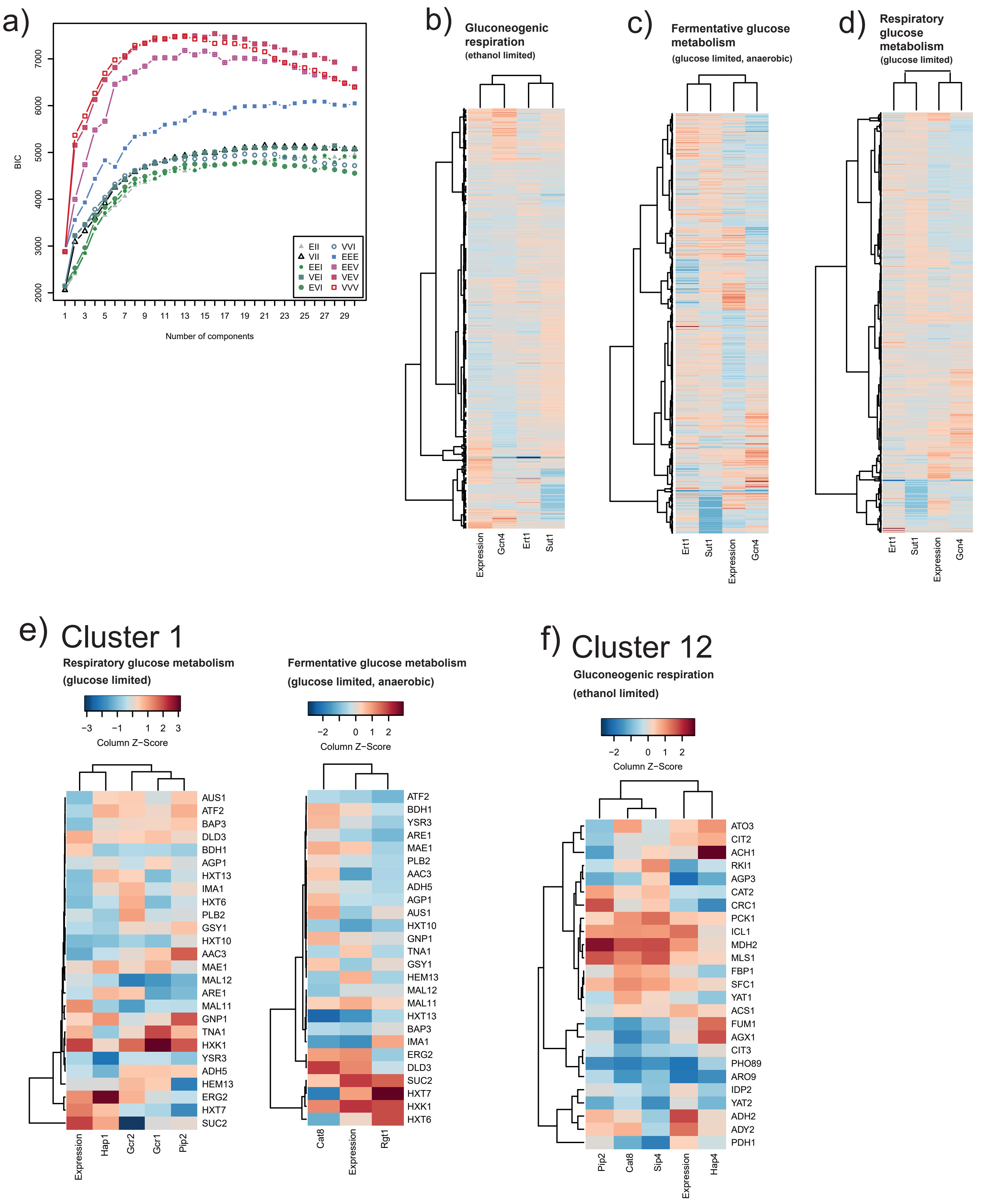

SUPPLEMENTARY FIGURE LEGENDS

Supplementary Figure 1: a) Six additional TFs that are included in further studies of the link between TF binding and transcript levels. For these TFs, the binding was previously described; Pip2, Oaf1, Ino2, Ino4 and Hap1 from Bergenholm et al, mSystems 2018 and Stb5 from Ouyang et al, FEBS YR 2018. The defined binding targets will differ slightly from the binding targets reported in those publications due to a newer version of GEM used to define peaks in this study. b) Peaks binned into 75bp segments at different positions relative to the TSS of all metabolic genes. TF-lines are labelled at their highest value relative to the y-axis. c) Comparison of the number of peaks for the different conditions. Red line indicates median and blue dot mean. Conditions were compared by a Wilcox test and p values FDR-adjusted for multiple testing. ** = $p < 0.01$, * = $p < 0.05$.

Supplementary Figure 2: a) Peaks from each condition were analyzed by MEME and the top significantly enriched DNA motif is reported. Below any enriched DNA motif is indicated the count of peaks and the E-value that MEME reports as a metric of how strongly enriched the motif is (lower is better). b) We compared our peak definition gene targets for the 8 TFs where Harbinson et al. Nature, 2004 reported peak definitions in multiple conditions. Where applicable, a comparison to gene targets as defined by MacIsaac et al. BMC Bioinformatics 2006 is also made. c-d) Deciding on the optimal region around the TSS to include TF binding signal from to predict transcript levels of the target gene. The black lines indicate the mean of all four conditions for a given interval of the promoters. MARS linear regressions was used to predict transcript levels from binding only in the indicated regions of all metabolic genes and the correlation of predicted transcript levels to real transcript levels indicated by R2.

Supplementary Figure 3: a) Plots illustrating the model building process for MARS in the four different conditions. The dotted red line indicates the size of the model (sum of TFs and spline rules) that are included at the maximum mean cross-validated out-of-fold R2, and this is the model that is used in Figure 2 b-e. g- b) Linear regression diagnostic plots of the models demonstrated in Figure 2 b-e. The top panels plots the residuals vs fitted values to look for linearity, the middle panels plot the distributions of residuals to look for normally distributed residuals and the bottom panels the distributions of residual size. c) Plots showing how the multiplied TF signals for the Ino2-Ino4 pair contributes in the linear regressions, as shown in Figure 3 e-h. The red lines and numbers show the coefficient of the multiplication term, meaning that higher red values indicates that this region of TF binding gives the strongest predicted transcriptional activation.

Supplementary Figure 4: a) Bayesian information criterion used to determine the optimal number of clusters in Figure 4. b-d) Heatmaps showing binding of Gcn4, Ert1 and Sut1 to

250 to 450 upstream of the TSS as well as expression levels over all metabolic genes in the three additional conditions, compared to Figure 6e. e-f) Heatmaps that, seen together with Figure 5, support the summary in Figure 6 f for glucose transport (panel e) and TCA cycle (panel f).

SUPPLEMENTARY DATA

Supplementary Data 1: Demonstration of ChIP-exo read start raw data. The read starts for each strand and replicate is loaded into IGV and a representative peak region is demonstrated.

Supplementary Data 2: List of all peaks, signal for different conditions and any motifs detected by GEM shown in Figure 1 and Supplementary Figure 1 a.

Supplementary Data 3: Genome-wide RNA sequencing read counts for all replicates of the four studied experimental conditions.

Supplementary Data 4: Total number of reads detected for in the TSS -500 to +500 promoter regions of all genes for the 21 TFs included in this study. The experimental conditions are in separate files.

Supplementary Data 5: Description of the selection criteria and process leading to the 21 TFs selected for this study.

Supplementary Data 6: R scripts used to process data and produce the figures.

Supplementary Data 7: Lists of genes defined as being in Group 1 or Group 2 in Figure 6 e.
