# Supplementary material for: Predictive models of eukaryotic transcriptional regulation reveals changes in transcription factor roles and promoter usage between metabolic conditions
Source: Nucleic Acids Res. 2019 Apr 12;47(10):4986–5000. doi: 10.1093/nar/gkz253 (PMC6547448; doi:10.1093/nar/gkz253)

**Supplementary data 1:**

**ChIP-exo raw data demonstration and replicate comparisons**

The following pages contain screenshots from IGV of raw data .wig files showing strand-specific read starts for all replicates. On the far left side is indicated which sample and replicate, where the second number in the file name indicates which sample, as follows:

| 1 | Aerobic fermentation replicate 1 |
| --- | --- |
| 2 | Aerobic fermentation replicate 2 |
| 3 | Gluconeogenic respiration replicate 1 |
| 4 | Gluconeogenic respiration replicate 2 |
| 5 | Fermentative glucose metabolism replicate 1 |
| 6 | Fermentative glucose metabolism replicate 2 |
| 7 | Respiratory glucose metabolism replicate 1 |
| 8 | Respiratory glucose metabolism replicate 2 |

Example: Cat8_1_3_plus.wig is showing all read starts on the plus strand for Cat8 in Gluconeogenic respiration replicate 1

The bottom two panels in IGV shows the location of any enriched motif, found by MEME and shown in Figure 1a and Supplementary Figure 1a.

**Cat8**

Peak at chr15, 83223. Promoter region of MDH2, YOL126C


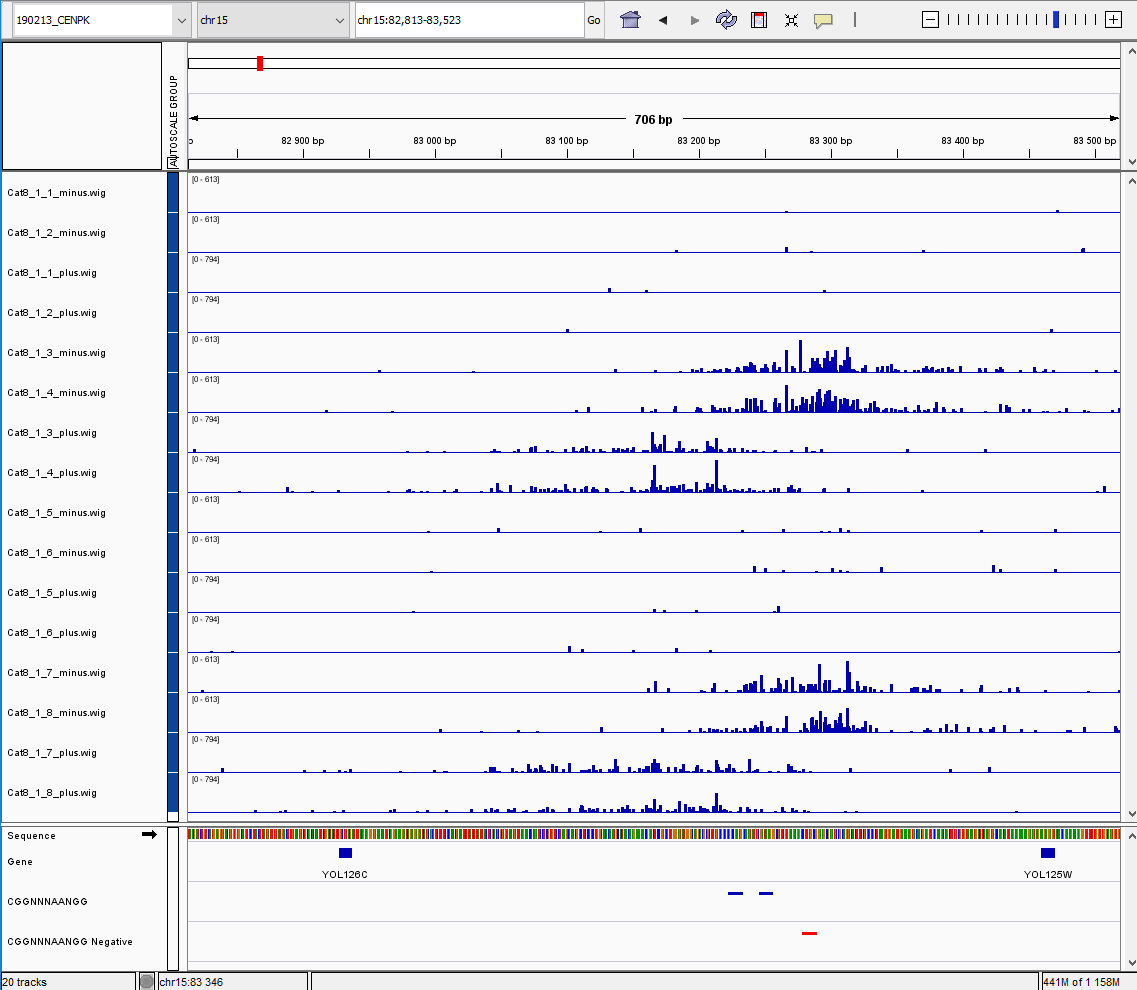


**Cbf1**

Peak at chr15, 511323. Promoter region of NUP1, YOR098C


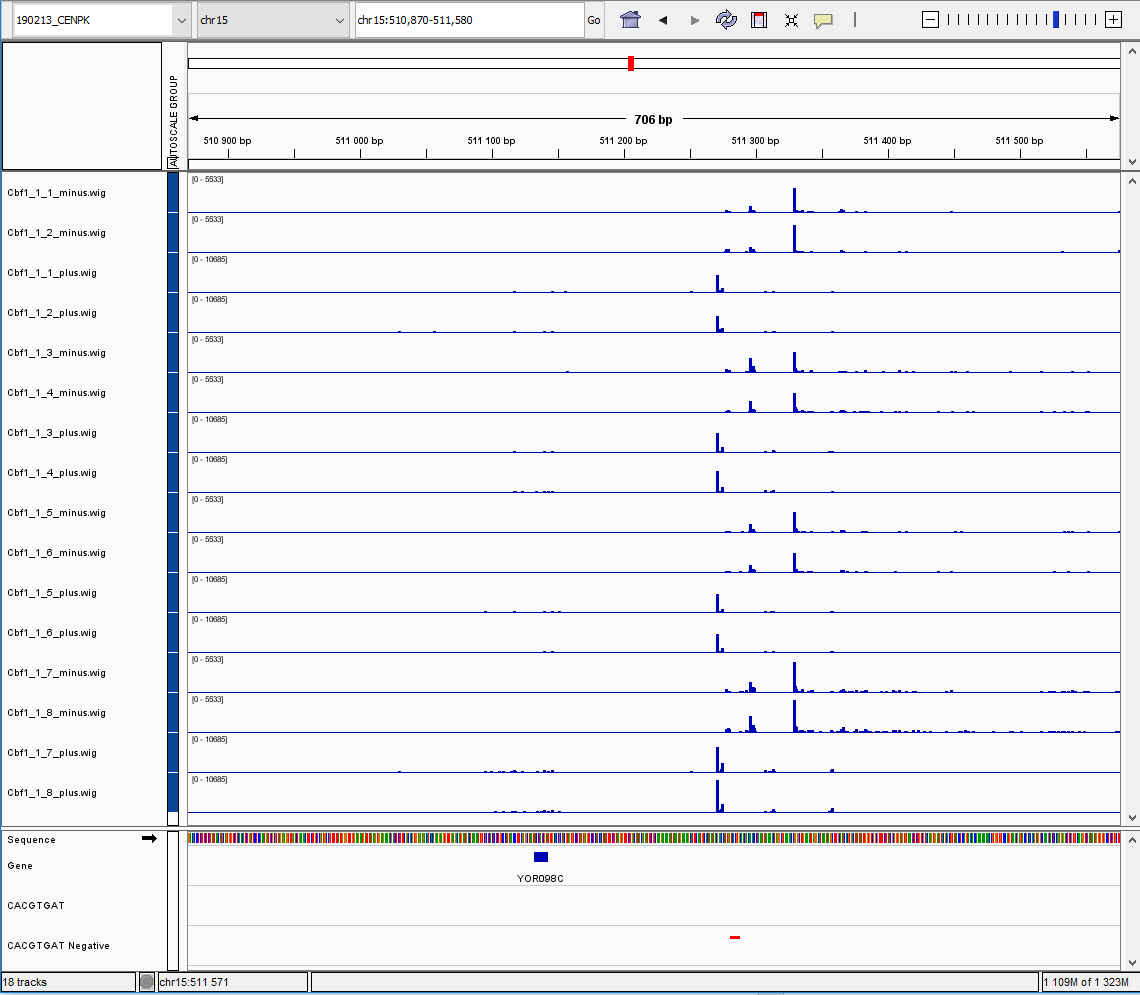


**Ert1**

Peak at chr 11, 636336. Promoter region of PCK1, YKR097W


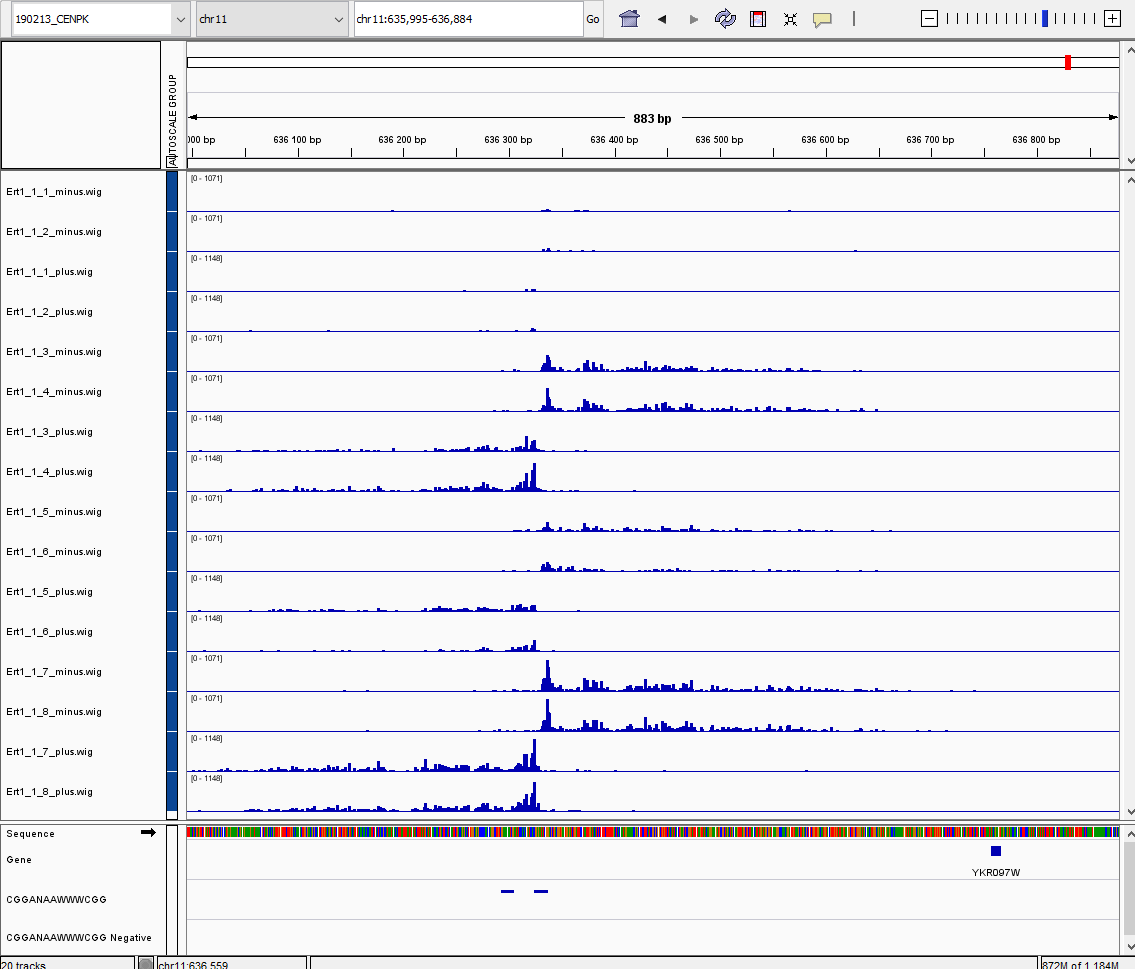


**Gcn4**

Peak at chr13, 367736. Promoter region of ARG7, YMR062C


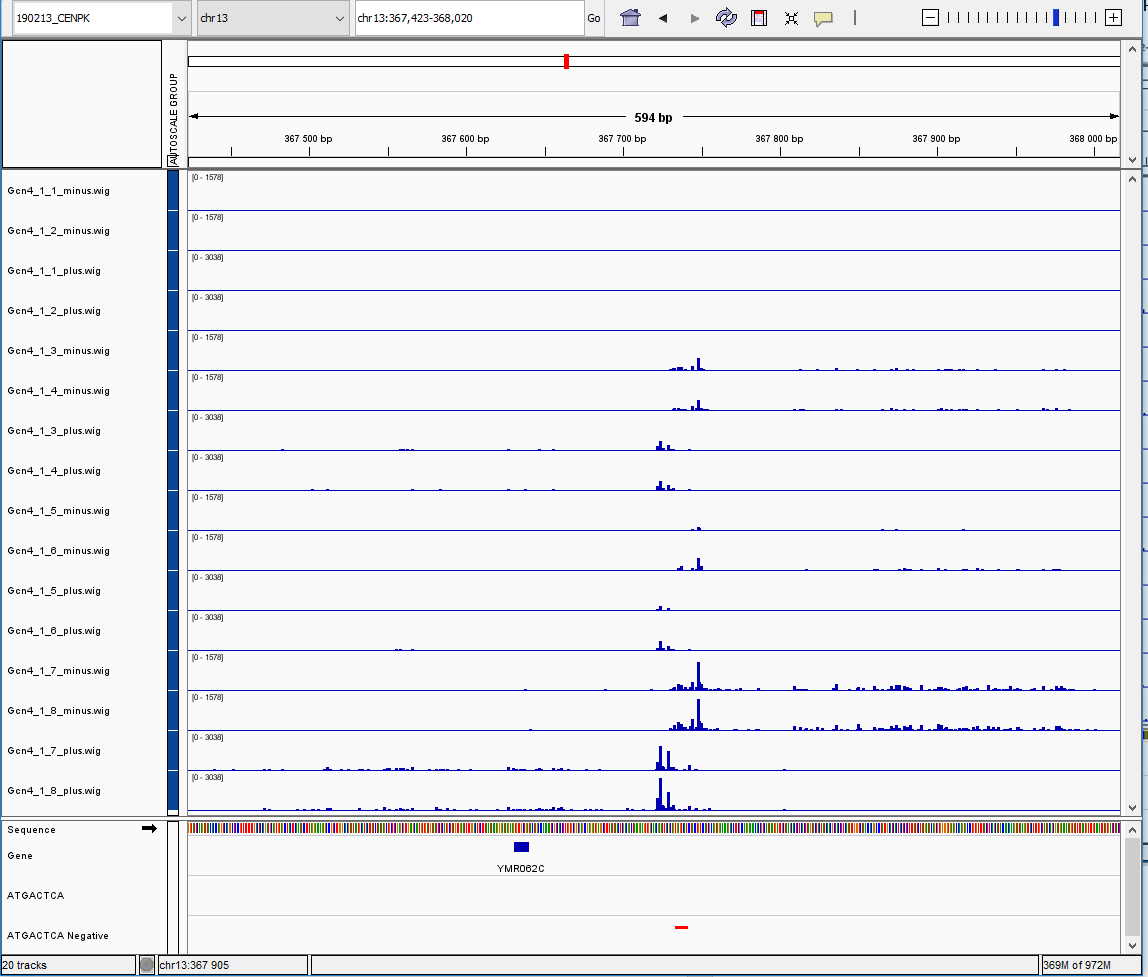


**Gcr1**

Peak at chr 13, 652652. Promoter region of PFK2, YMR205C


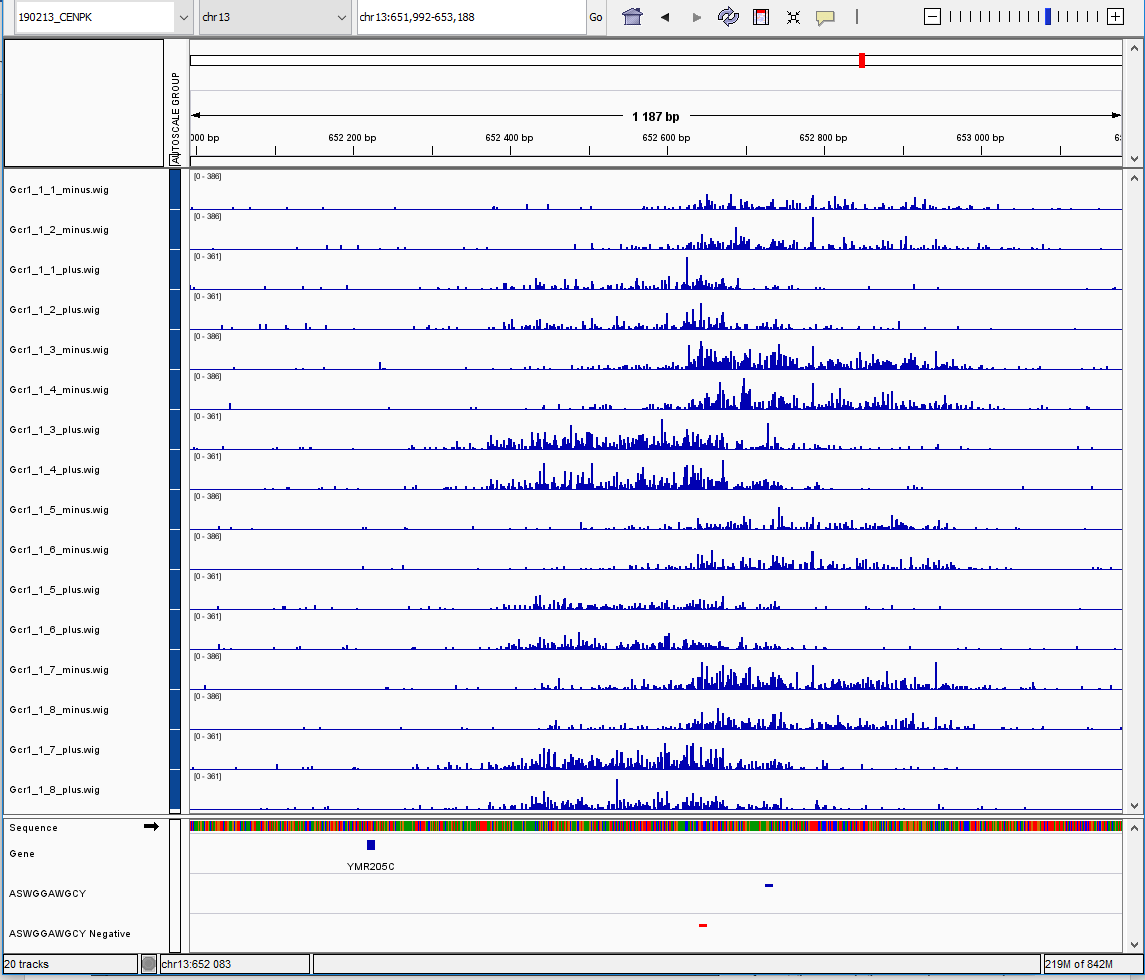


**Gcr2**

Peak at chromosome 11, position 333866. Promoter region of FBA1, YKL060C


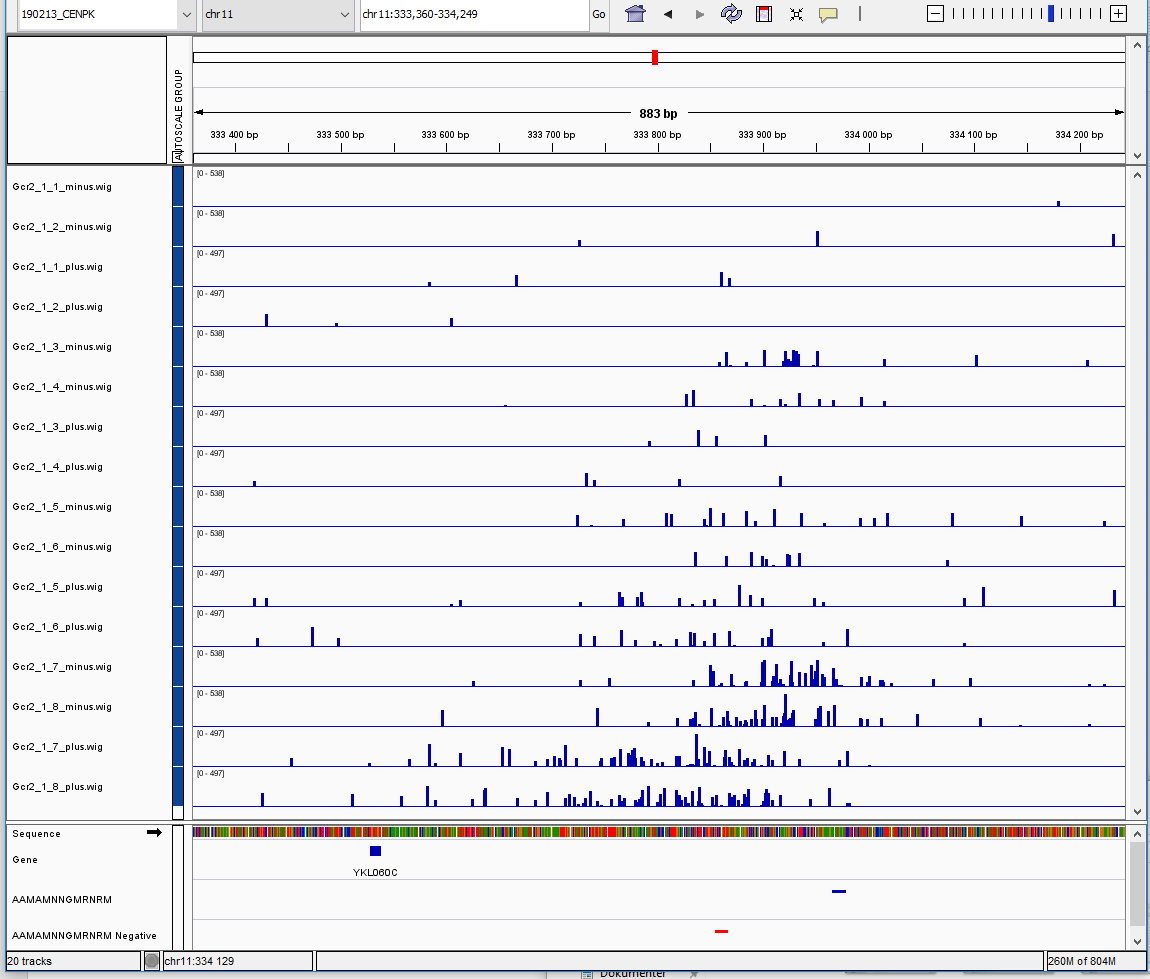


**Hap4**

Peak at chromosome 13, position 85802. Promoter region of RPM2, YML091C


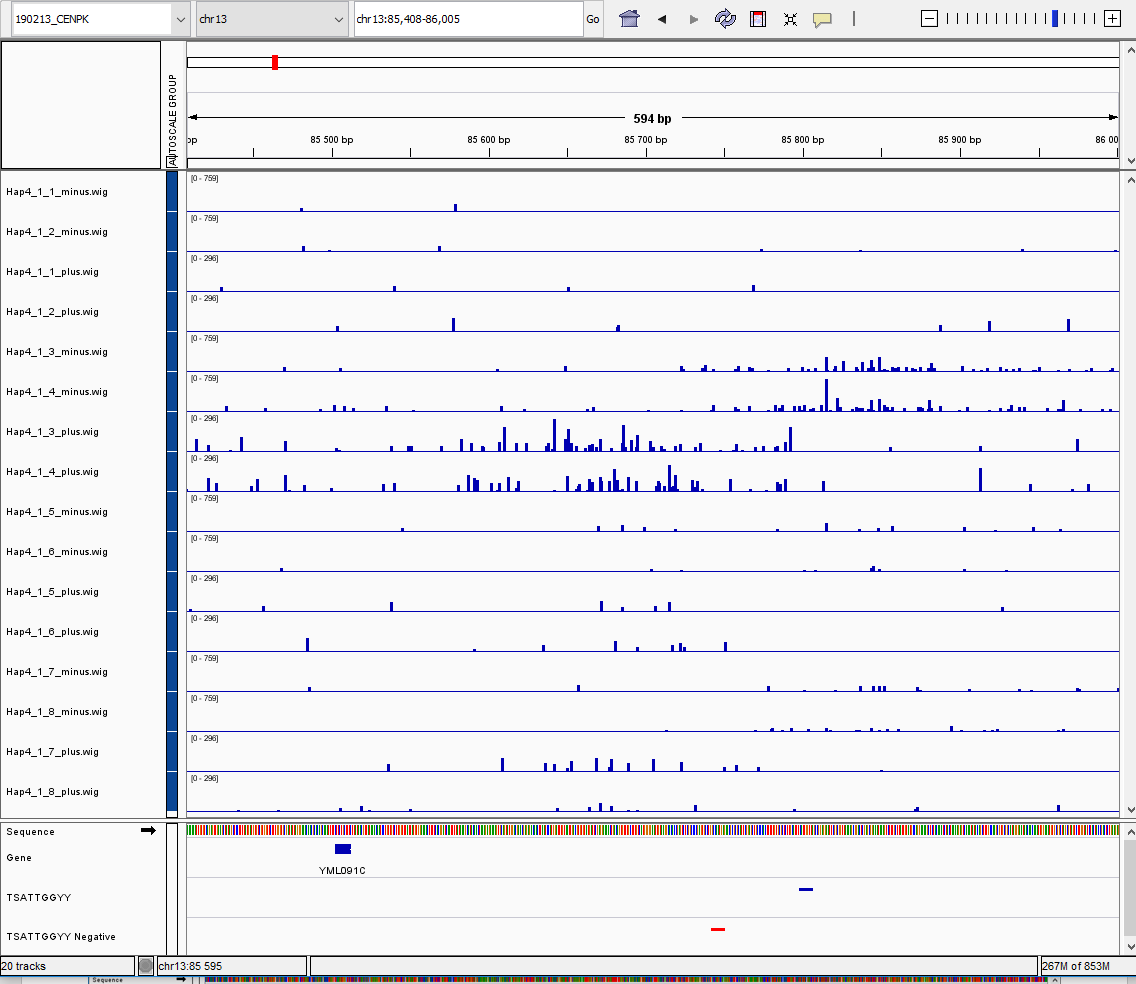


**Leu3**

Peak at chromosome 15, position 1037680. Promoter region of GDH1, YOR375C


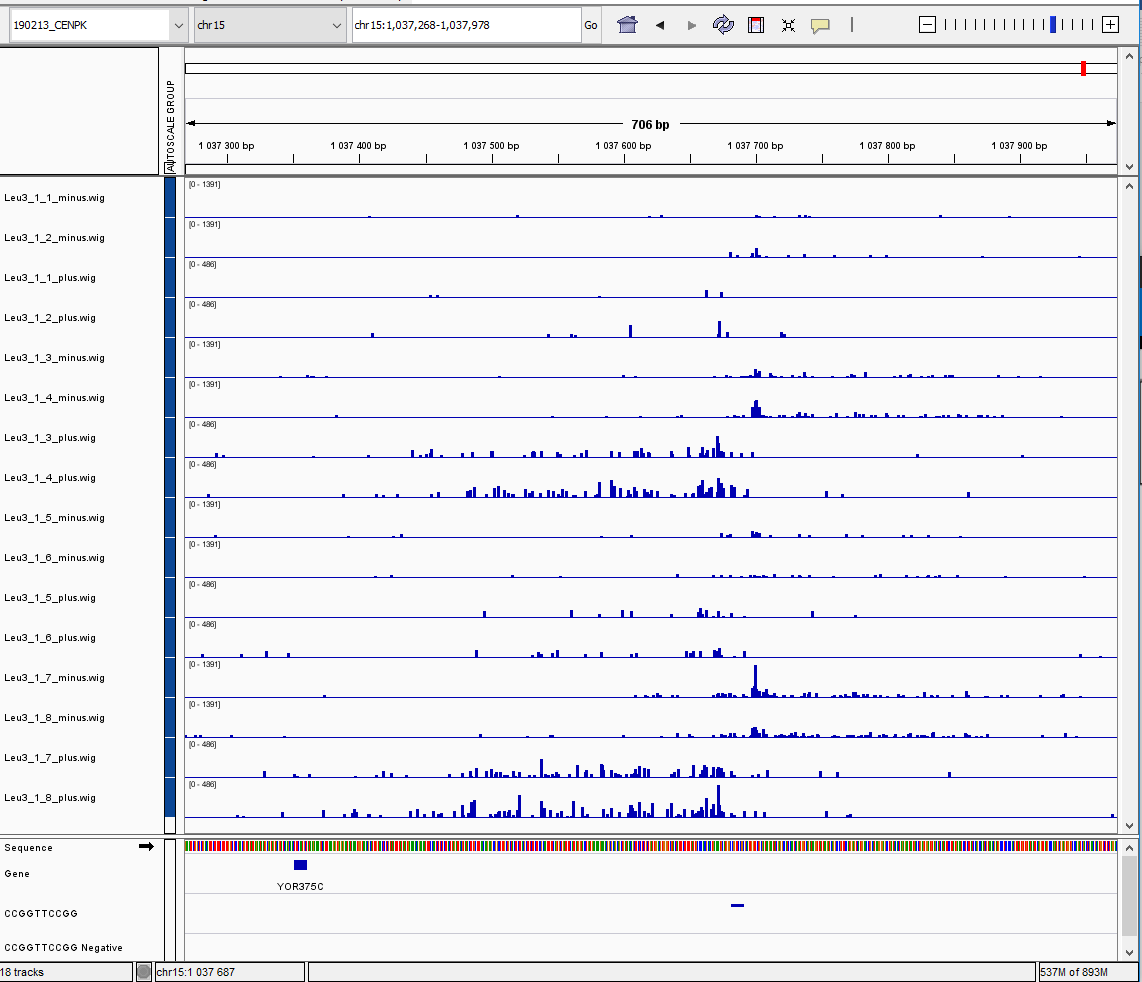


**Rds2**

Peak at chromosome 11, position 636326. Promoter region of PCK1, YKR097W


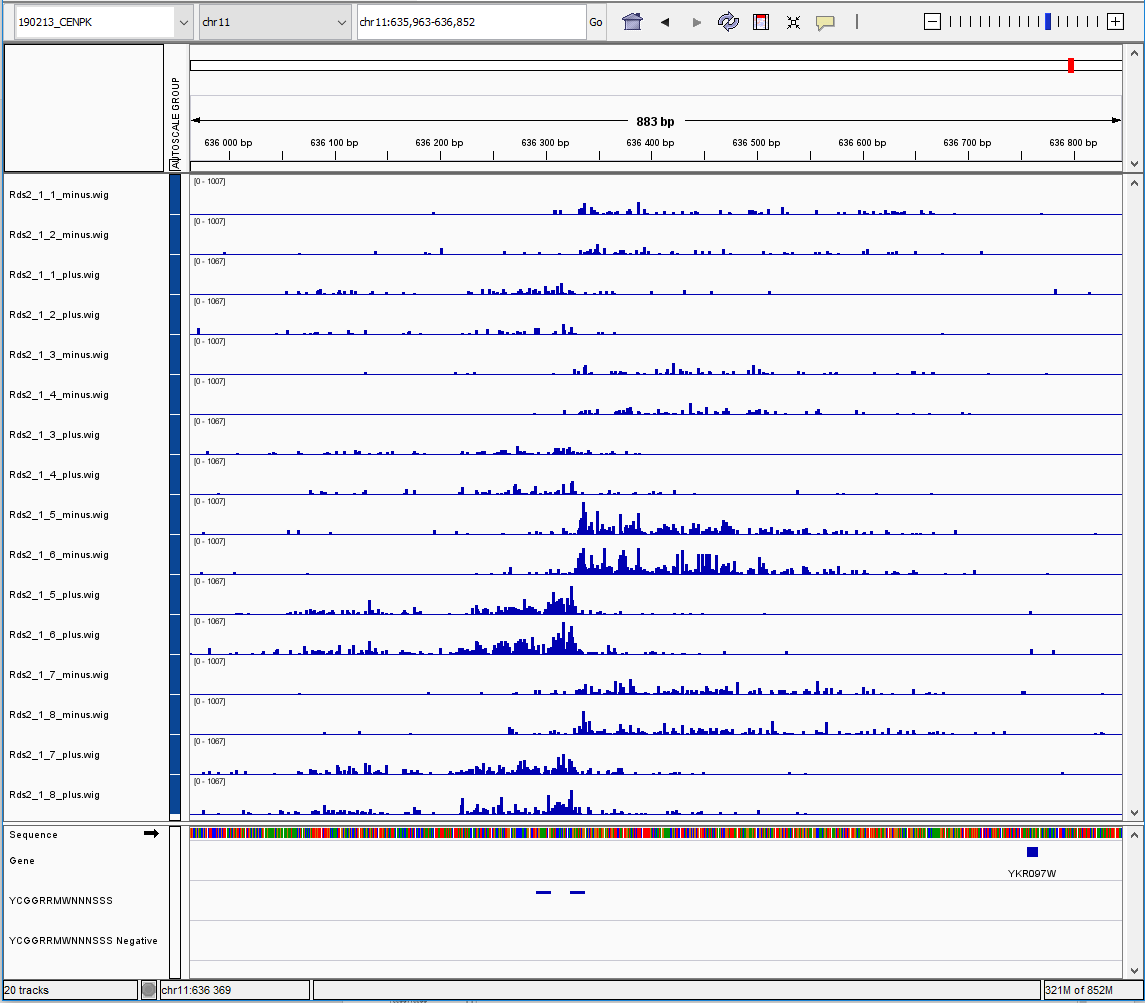


**Rgt1**

Peak at chromosome 13, position 270411. Promoter region of HXT2, YMR011W


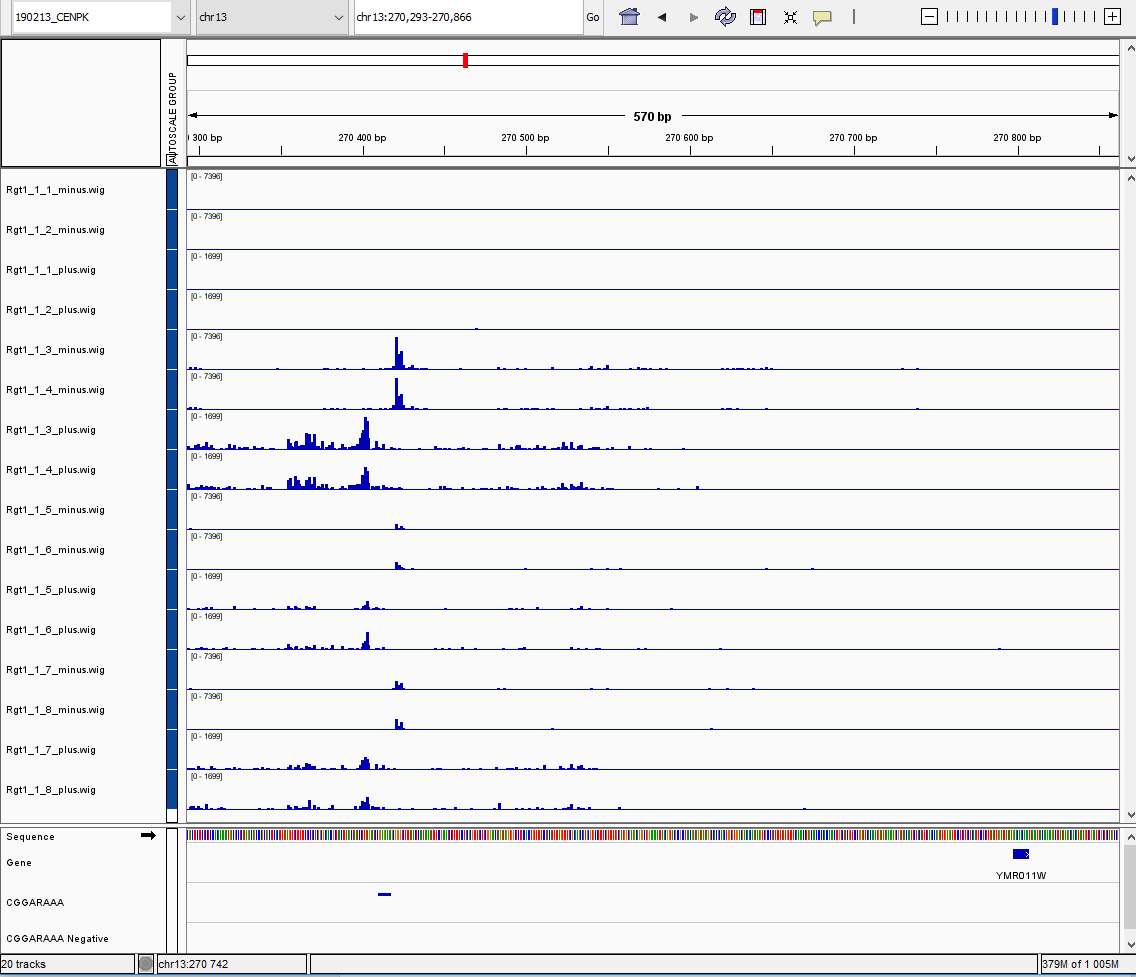


**Rtg1**

Peak at chromosome 12, position 697547. Promoter region of ACO1, YLR304C


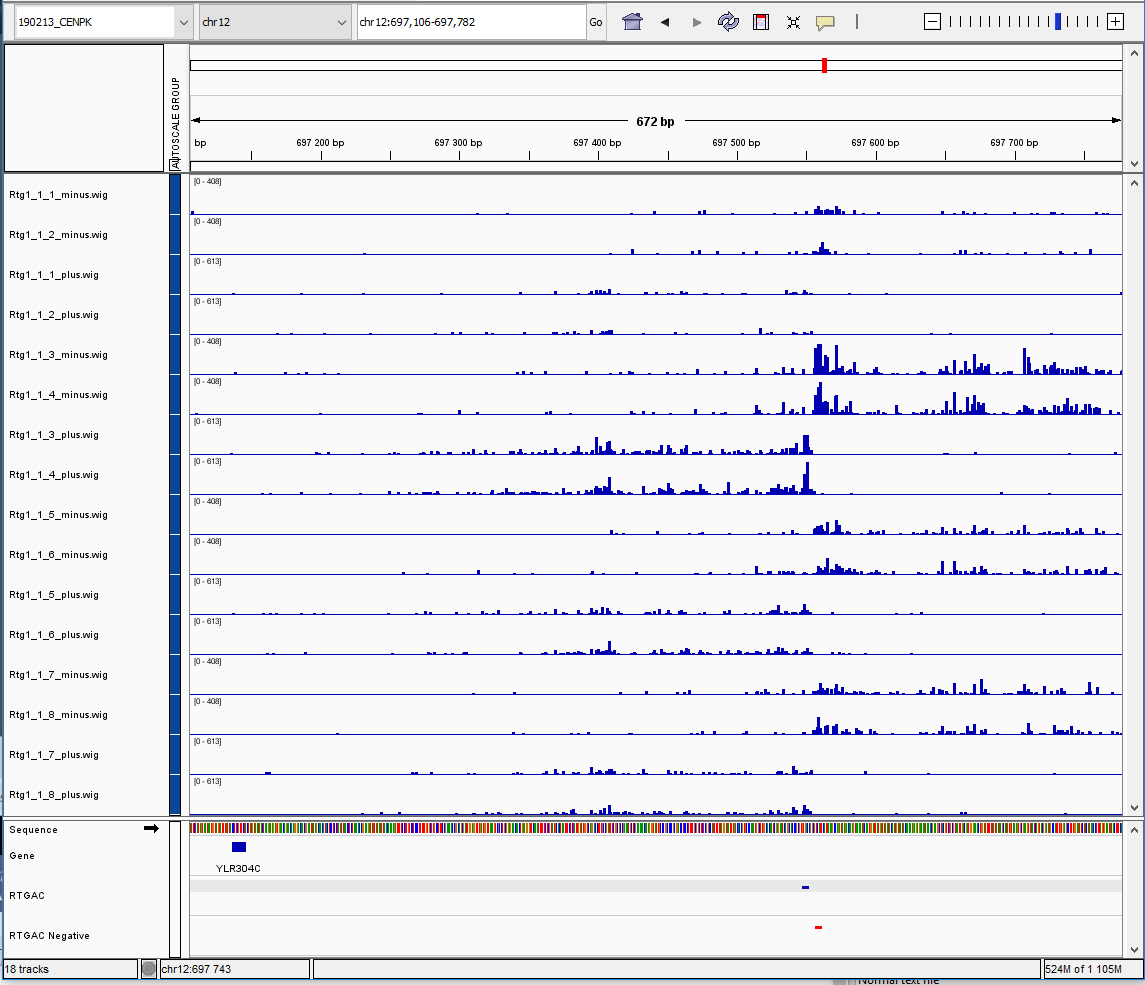


**Rtg3**

Peak at chromosome 5, position 17163. Promoter region of DLD3, YEL071W


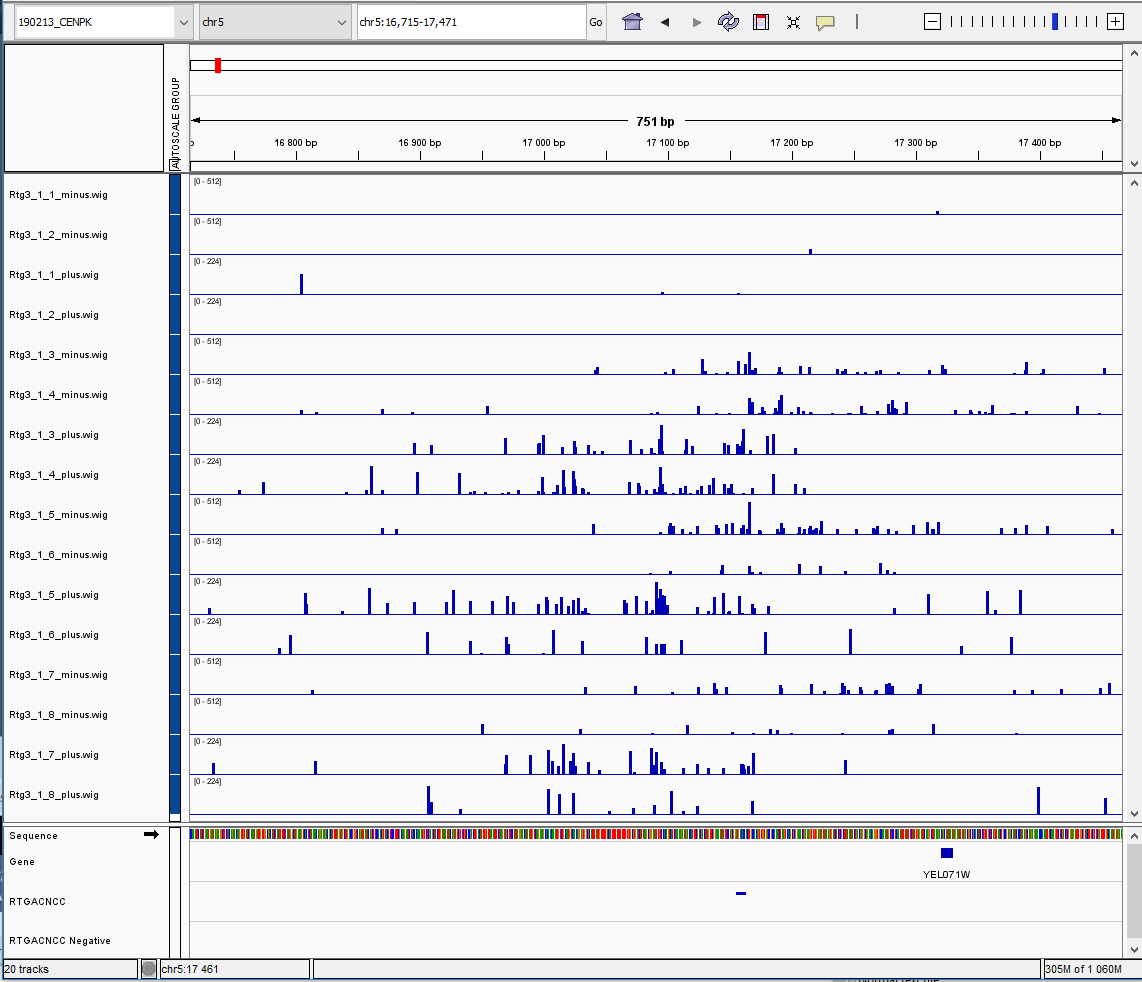


**Sip4**

Peak at chromosome 11, position 636385. Promoter region of PCK1, YKR097W


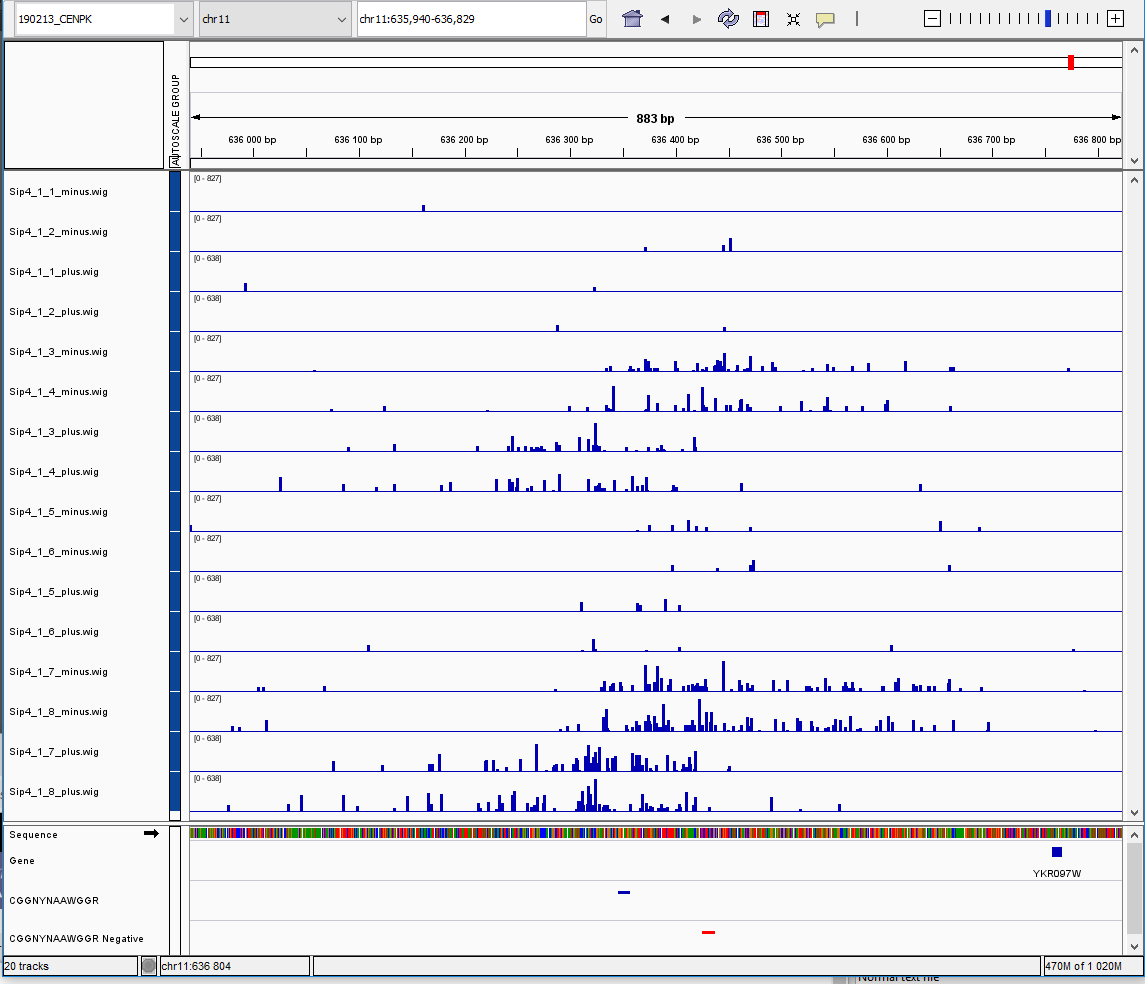


**Sut1**

Peak at chromosome 11, position 237790. Promoter region of HAP4, YKL109W


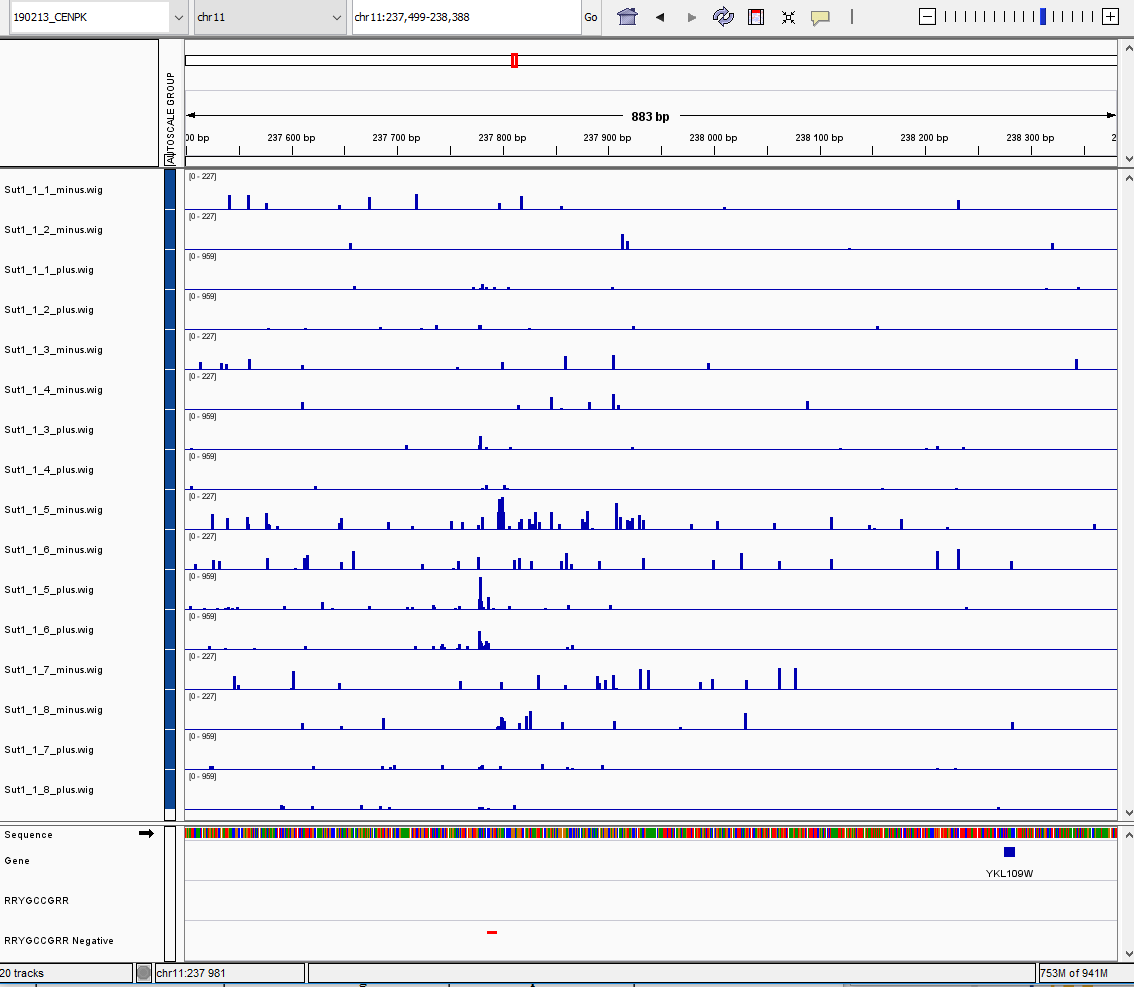


**Tye7**

Peak at chromosome 8, position 451338. Promoter region of ENO2, YHR174W


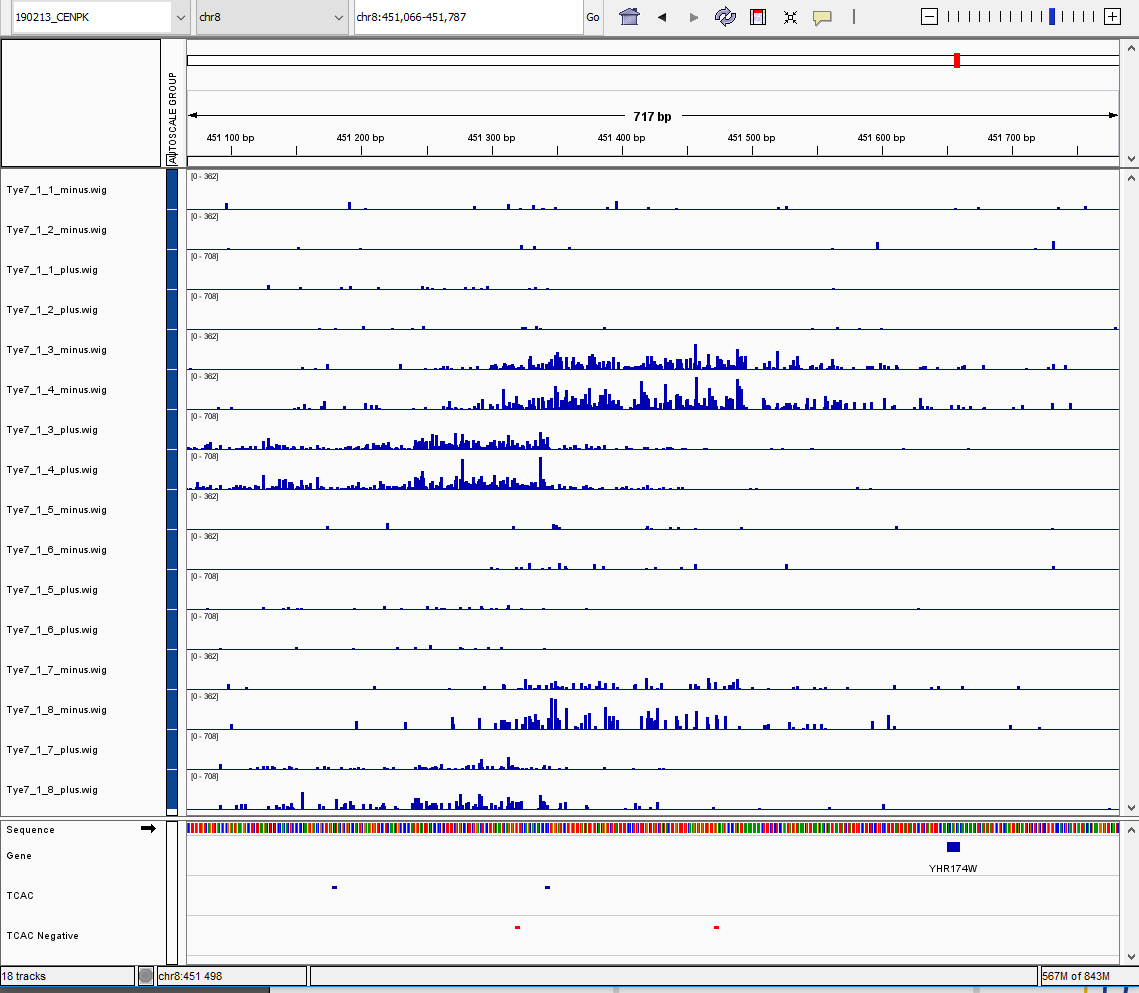

Supplement: gkz253_Supplemental_Files [file gkz253_supplemental_files.zip › Suppl_Data_1_Raw_data.docx]
