# Supplementary material for: Predictive models of eukaryotic transcriptional regulation reveals changes in transcription factor roles and promoter usage between metabolic conditions
Source: Nucleic Acids Res. 2019 Apr 12;47(10):4986–5000. doi: 10.1093/nar/gkz253 (PMC6547448; doi:10.1093/nar/gkz253)
Supplement: gkz253_Supplemental_Files [file gkz253_supplemental_files.zip › Suppl_Data_5_TF_selection.docx]

**Supplementary Data 1 – TF selection**

| **TF** | **Total count of targeted genes** | **Fraction of targeted genes in CCM** |
| --- | --- | --- |
| Hap4 | 112 | 19% |
| Gcr2 | 58 | 14% |
| Tye7 | 86 | 12% |
| Sut1 | 125 | 10% |
| Hap1 | 192 | 9% |
| Stb5 | 76 | 9% |
| Ino4 | 123 | 9% |
| Ino2 | 91 | 9% |
| Leu3 | 52 | 8% |
| Hap3 | 52 | 8% |
| Pip2 | 83 | 7% |
| Cbf1 | 73 | 7% |
| Cin5 | 235 | 7% |
| Gcn4 | 103 | 7% |
| Oaf1 | 117 | 5% |
| Sko1 | 59 | 5% |

Table 1: All TFs with >50 targets and >5% enrichment to central carbon metabolism (CCM) enzymes in the Harbison et al YPD data (1). TFs in green passed validation for binding of the tagged TF to a known target by ChIP-qPCR while those in red did not pass validation.

| **TF** | **Justification for including** | **Reference** | **< 50 targets** | **< 5% CCM** | **Not studied** |
| --- | --- | --- | --- | --- | --- |
| Gcr1 | Major glycolytic regulator | (2) | X |  |  |
| Rgt1 | Regulator of glucose import | (3) | X |  |  |
| Rtg1 | Proposed to coordinate CCM fluxes in several pathways | (4) |  | X |  |
| Rtg3 | Proposed to coordinate CCM fluxes in several pathways | (4) |  | X |  |
| Sip4 | Gluconeogenesis regulator during growth on ethanol | (5) | X |  |  |
| Cat8 | Gluconeogenesis regulator during growth on ethanol | (6) |  |  | X |
| Ert1 | Proposed to be essential for metabolic changes in the diauxic shift | (7) |  |  | X |
| Rds2 | Proposed regulator of gluconeogenesis | (8) |  |  | X |

Table 2: TFs included in this study that were either not studied by Harbison et al or not above the thresholds set in Table 1. The three right-most columns indicate their status in the Harbison dataset.

**References**

1. Harbison,C.T., Gordon,D.B., Lee,T.I., Rinaldi,N.J., Macisaac,K.D., Danford,T.W., Hannett,N.M., Tagne,J.B., Reynolds,D.B., Yoo,J., *et al.* (2004) Transcriptional regulatory code of a eukaryotic genome. *Nature*, **431**, 1–5.

2. Baker,H. V (2006) GCR1 of Saccharomyces cerevisiae encodes a DNA binding protein whose binding is abolished by mutations in the CTTCC sequence motif. *Proc. Natl. Acad. Sci.*, **88**, 9443–9447.

3. Ozcan,S., Leong,T. and Johnston,M. (1996) Rgt1p of Saccharomyces cerevisiae, a key regulator of glucose-induced genes, is both an activator and a repressor of transcription. *Mol. Cell. Biol.*, **16**, 6419–6426.

4. Fendt,S.-M., Oliveira,A.P., Christen,S., Picotti,P., Dechant,R.C. and Sauer,U. (2010) Unraveling condition-dependent networks of transcription factors that control metabolic pathway activity in yeast. *Mol. Syst. Biol.*, **6**, 432.

5. Hiesinger,M., Roth,S., Meissner,E. and Schüller,H.J. (2001) Contribution of Cat8 and Sip4 to the transcriptional activation of yeast gluconeogenic genes by carbon source-responsive elements. *Curr. Genet.*, **39**, 68–76.

6. Hedges,D., Proft,M. and Entian,K.D. (2015) CAT8, a new zinc cluster-encoding gene necessary for derepression of gluconeogenic enzymes in the yeast Saccharomyces cerevisiae. *Mol. Cell. Biol.*, **15**, 1915–1922.

7. Gasmi,N., Jacques,P.E., Klimova,N., Guo,X., Ricciardi,A., Robert,F. and Turcotte,B. (2014) The switch from fermentation to respiration in Saccharomyces cerevisiae is regulated by the Ert1 transcriptional activator/repressor. *Genetics*, **198**, 547–560.

8. Robert,F., Drouin,S., Larochelle,M., Soontorngun,N. and Turcotte,B. (2007) Regulation of Gluconeogenesis in Saccharomyces cerevisiae Is Mediated by Activator and Repressor Functions of Rds2. *Mol. Cell. Biol.*, **27**, 7895–7905.
